# *Bupleurum marginatum* Wall.ex DC in Liver Fibrosis: Pharmacological Evaluation, Differential Proteomics, and Network Pharmacology

**DOI:** 10.3389/fphar.2018.00524

**Published:** 2018-05-17

**Authors:** Xiujie Liu, Yu Shi, Yinghui Hu, Ke Luo, Ying Guo, Weiwei Meng, Yulin Deng, Rongji Dai

**Affiliations:** ^1^School of Life Science, Institute of Space Biology and Medical Engineering, Beijing Institute of Technology, Beijing, China; ^2^School of Basic Medical Sciences, Shanxi Medical University, Taiyuan, China; ^3^School of Life Science, Beijing Institute of Technology, Beijing, China

**Keywords:** *Bupleurum marginatum* Wall.ex DC, liver fibrosis, network pharmacology, differential proteomics, bioactive ingredients

## Abstract

Liver fibrosis is a common pathological feature of many chronic liver diseases. *Bupleurum marginatum* Wall.ex DC (ZYCH) is a promising therapeutic for liver fibrosis. In this study, 25 compounds were isolated from ZYCH, and the effects of ZYCH on DMN-induced liver fibrosis in rats were evaluated. The optimal effect group (H-ZYCH group) was selected for further proteomic analysis, and 282 proteins were altered in comparison to the DMN model group (FC > 1.2 or < 0.83, *p* < 0.05). Based on GO annotation analysis, clusters of drug metabolism, oxidative stress, biomolecular synthesis and metabolism, positive regulation of cell growth, extracellular matrix deposition, and focal adhesion were significantly regulated. Then networks of the altered proteins and compounds was generated by Cytoscape. Importantly, triterpenoid saponins and lignans had possessed high libdock scores, numerous targets, important network positions, and strong inhibitory activity. These findings may suggest that triterpenoid saponins and lignans are important active compounds of ZYCH in liver fibrosis and targeted by proteins involved in liver fibrosis. The combination of network pharmacology with proteomic analysis may provide a forceful tool for exploring the effect mechanism of TCM and identifying bioactive ingredients and their targets.

## Introduction

Liver fibrosis is the self-repair compensatory response of hepatocytes after damage, which can impact the interchange of material between hepatocytes and blood, leading to internal and external circulatory disturbances (Trautwein et al., 2015). Liver fibrosis is a common pathological feature of various chronic liver diseases (Iredale et al., 2017), and it may develop to liver cirrhosis if it couldn't been diagnosed and treated promptly (Luetkemeyer and Wyles, 2017). Recently, clinical studies have shown that liver fibrosis could be reversed (Zoubek et al., 2017). Effective liver fibrosis therapies were mainly based on the original causes of the disease, such as anti-inflammatory treatment (Oró et al., 2016), inhibiting intrahepatic ECM deposition and promoting ECM degradation (Prestigiacomo et al., 2017). Over the past 10 years, anti-liver fibrosis treatment research has made great progress in traditional Chinese medicine (TCM), such as Chinese herbal prescription medicine [Fuzheng Huayu Formula (Chen et al., 2016), Biejia Ruangan tablets (Dong et al., 2016), herbal compound 861 (Hou et al., 2016)] and effective components of TCM [silymarin (Clichici et al., 2016), saikosaponins (Fan et al., 2007)]. Significant differences were observed in different TCM for anti-liver fibrosis (Du and You, 2001), but the main advantages of TCM are focused on the comprehensive action under the guidance of TCM therapeutic thinking (Yang et al., 2000). In this regard, comprehensive pharmacological effects of multi-levels, multi-pathways, and multi-targets is the characteristic of multi-components TCM for anti-liver fibrosis, which has obvious advantages and broad application prospects.

*Bupleurum marginatum* Wall.ex DC (Zhuyechaihu, ZYCH) is a dry whole plant of Apiales, Umbellales, which is commonly used in drugs for hepatitis, liver depression, and qi stagnation in Dai medicine (Lu et al., 2016). ZYCH is also one of the basic remedy of the anti-liver fibrosis TCM formula “Baogan capsule”(Liu et al., 2017b). At present, few studies have focused on ZYCH in China and abroad. The main chemical components of ZYCH are saikosaponins, flavonoids, sterols, and lignans. In previous studies, 29 compounds were isolated from the herb, including 6 saikosaponins (Liang et al., 2003), 5 flavonoids (Liang et al., 2003; Wang et al., 2007), 7 sterols (Liang et al., 2003; Wang et al., 2007), 5 lignans (Liu et al., 2008; Ashour et al., 2012), 3 lactones (Liang et al., 2003), 2 sugar alcohols (Liang et al., 2003), and 1 fatty acid (Wang et al., 2007). Pharmacological activity studies showed that the volatile oil part of ZYCH with strong anti-inflammatory activity accelerates tumor cells apoptosis and inhibits tumor cells growth (Ashour et al., 2009). In a follow-up report, using methylene chloride and methanol extract of ZYCH, an anti-infective and anti-tumor effect were also observed (Ashour et al., 2014). Recently, the study on ZYCH treating liver fibrosis had not be reported.

Network pharmacology is a developing field based on systems pharmacology (Hu et al., 2014). Text mining assays and molecular docking involved in network pharmacology are available for analyzing interactions between compounds and proteins. Network pharmacology has been applied to reveal the molecular mechanisms of TCM on complicated diseases (Wu and Wu, 2015), could provide a holistic understanding of TCM pharmacological molecular mechanisms. In previous study, the underlying action mechanism of Wu-Tou decoction (WTD) in rheumatoid arthritis (RA) (Guo et al., 2017) and Xipayi Kui Jie'an (KJA) in ulcerative colitis (UC) (Yu et al., 2017) were explored using network pharmacology.

In this study, 25 compounds were separated and identified, of which 21 compounds were isolated from ZYCH for the first time. The effect of ZYCH on DMN-induced liver fibrosis rats was investigated, and then the liver altered proteins between optimal effect group (H-ZYCH) and model group were found out using proteomics approaches. The interaction between the compounds and the altered proteins was found out based on molecular docking, then the compounds-targets network of ZYCH anti-liver fibrosis was constructed. This study aims to elucidate the pharmacological effective of ZYCH on liver fibrosis, and it may provide guidelines for exploring effect mechanisms of TCM and identifying the active compounds and key targets.

## Materials and methods

### General experimental procedures

The ESI-MS spectra were recorded on an LTQ orbitrap ETD equipped with Easy-nLC 1000 (Thermo Fisher, MA, USA). The HR-ESI-MS spectra were obtained on an Agilent 6210 TOF LC mass spectrometer (Agilent, Santa Clara, USA). The 1D and 2D NMR were measured on a Bruker AVANCE DRX-500 spectrometer (Bruker, Karlsruhe, Germany) using methanol-d_4_ and acetone-d_6_ as the solvent, with tetramethylsilane (TMS) as an internal reference. Analytical HPLC was performed on a Shimadzu LC-20AD chromatograph (Shimadzu, Kyoto, Japan) equipped with the YMC ODS-A C18 reversed-phase column (Cosmosil, 5 μm, 250 × 4.6 mm) (YMC, Kyoto, Japan). Semi-preparative high-performance liquid chromatography (HPLC) was performed on a Shimadzu LC-20AD chromatograph (Shimadzu, Kyoto, Japan) equipped with a YMC ODS-A C18 reversed-phase column (Cosmosil, 5 μm, 250 × 20 mm) (YMC, Kyoto, Japan). Normal phase silica gel (200–300 mesh, Qingdao Haiyang Chemical Co., Ltd.) and octadecylsilanized (ODS) silica gel (50 μm, YMC Ltd., Japan) were also used for column chromatography (CC). Bioactivity was measured in a Thermo Electron 3543 CO_2_ incubator (Thermo Electron, Massachusetts, USA) and a Labsystems Multiskan MS microplate reader (Thermo Electron, Massachusetts, USA). Alanine aminotransferase (ALT), aspartate aminotransferase (AST), albumin (Alb), alkaline phosphatase (AKP) and total bilirubin (TBIL), superoxide dismutase (SOD), malondialdehyde (MDA), hydroxyproline (Hyp), tumor necrosis factor-α (TNF-α), and collagen type IV (COLIV) kits were obtained from Nanjing Jiancheng Bioengineering Research Institute (Nanjing, China). α-smooth muscle actin(α-SMA, Enzyme-linked Biotechnology, Shanghai, China) was also used as kits. TGF-β1 was obtained from PeproTech (Rocky Hill, NJ, USA), and the iTRAQ Reagent-8 plex Multiplex Kit (Applied Biosystems, CA, USA) was using for labeling. Ligalong (Silymarin capsules, SC) was manufactured by Madaus AG (Cologne, Germany).

### Extraction and isolation of ZYCH

The quality of ZYCH was kindly provided by Dai hospital of Xishuangbanna (Yunnan, China). The voucher specimen (NO. YN20130561) was deposited in Beijing BIT&GY Pharmaceutical R&D co., Ltd.

The air-dried and powdered whole plant samples of ZYCH (17 kg) were extracted three times with 170 L 75% ethanol (5% ammonium hydroxide) under reflux. The crude extract (2782 g) was dried under reduced pressure, and 500 g was removed for later. The remainder was suspended in water and successively treated with petroleum ether (PE), ethyl acetate (EtOAc), and n-butanol (n-BuOH). Each extraction part was dried at 60°C and stored in an airtight container at−80°C prior to further analysis.

The EtOAc fraction was applied to a silica gel column (200-300 mesh) and eluted successively with PE–EtOAc (4:1, 1:1, 1:8, 1:20, 0:100) to obtain 5 fractions (EA-Fr.1~ EA-Fr.5). EA-Fr.4 was subjected to a silica gel column (200-300 mesh) and eluted with PE–EtOAc 2:1 to afford 5 subfractions (EA-Fr.4.1~ EA-Fr.4.5) according to TLC. EA-Fr.4.3 was further purified by preparative HPLC (ACN-H_2_O, 60:40) to afford compound **1**. EA-Fr.4.4 was further purified by preparative HPLC (ACN-H_2_O, 60:40) to afford compounds **2**, **3**, **4**.

The n-BuOH fraction was extracted with acetone for separation into the acetone soluble fraction (n-BuOH-DMK fraction) and acetone insoluble fraction (n-BuOH-n-BuOH fraction). The n-BuOH-DMK fraction was applied to a silica gel column (200-300 mesh) and eluted successively with EtOAc-MeOH (100:1, 75:1, 25:1, 0:100) to obtain 4 fractions (DMK-Fr.1~ DMK-Fr.4). DMK-Fr.2 was subjected to a silica gel column (200-300 mesh) and eluted with CYH-DMK (4:1, 3:2, 1:2, 0:100) to afford 4 subfractions (DMK-Fr.2.1~DMK-Fr.2.4). DMK-Fr.2.2 was further purified by preparative HPLC (ACN-H_2_O, 55:45) to afford compound **5**, **6**, **7**. DMK-Fr.2.3 was further purified by preparative HPLC (ACN-H_2_O, 45:55) to afford compounds **8**, **9**, **10**.

The n-BuOH-n-BuOH fraction was applied to a silica gel column (200-300 mesh) and eluted successively with EtOAc-MeOH (25:1, 4:1, 1:1, 0:100) to obtain 4 fractions (n-BuOH-Fr.1~ n-BuOH-Fr.4). n-BuOH-Fr.1 was subjected to a silica gel column (200-300 mesh) and eluted with EtOAc-MeOH, 3:4 to afford 4 subfractions (n-BuOH-Fr.1.1~ n-BuOH-Fr.1.4) according to TLC. The n-BuOH-Fr.1.1 was further purified by preparative HPLC (ACN-H_2_O, 35:65) to produce compound **11**. n-BuOH-Fr.1.2 was further purified by preparative HPLC (ACN-H_2_O, 35:65) to produce compounds **12**, **13**. n-BuOH-Fr.1.3 was further purified by preparative HPLC (ACN-H_2_O, 35:65) to produce compounds **14**, **15**. n-BuOH-Fr.1.4 was further purified by preparative HPLC (ACN-H_2_O, 30:70) to produce compounds **16**, **17**, **18**, **19**. The n-BuOH-Fr.2 was subjected to a silica gel column (200-300 mesh) eluted with EtOAc-MeOH, 2:3 to afford 5 subfractions (n-BuOH-Fr.2.1~ n-BuOH-Fr.2.5) according to TLC. n-BuOH-Fr.2.1 was further purified by preparative HPLC (ACN-H_2_O, 30:70) to produce compound **20**. n-BuOH-Fr.2.2 was further purified by preparative HPLC (ACN-H_2_O, 30:70) to produce compound **21**. n-BuOH-Fr.2.3 was further purified by preparative HPLC (ACN-H_2_O, 28:72) to produce compounds **22**, **23**, **24**, **25**.

### Effect of ZYCH on rat liver fibrosis

#### Animals

Male Sprague-Dawley rats (SPF degree, aged 6-8 weeks and weighing 180-200 g) were obtained from the Laboratory Animal Center (Academy of Military Medical Sciences, Beijing, China). The rats were maintained under temperature-controlled conditions at 25 ± 2°C with 40-60% relative humidity and a 12 h light/dark cycle. The rats in the experiment had *ad libitum* access to food and water. The animals were acclimatized for 1 week. According to the dosing regimen (Supplementary Table 1), a total of 70 rats were randomly divided into seven groups of 10 rats per group, including the control group (*n* = 10), DMN model group (*n* = 9), silymarin capsules group (SC group, *n* = 10), low-dose ZYCH group (L-ZYCH group, *n* = 10), middle-dose ZYCH group (M-ZYCH group, *n* = 10), high-dose ZYCH group (H-ZYCH group, *n* = 9), and higher-dose ZYCH group (HR-ZYCH group, *n* = 9). In this study, SC was used as positive control. The rats were intraperitoneally injected with DMN at 10 mg/kg body weight 3 consecutive days per week for 4 weeks, excluding the control group (Liu et al., 2017b). The experimental rats in the different groups were fed with ZYCH / SC for 4 weeks through oral administration from the first week of DMN exposure. After the 4-week treatment period, the rats were immediately sacrificed under anesthesia after an overnight fast. Then, serum and liver samples were collected for further analysis (Tabet et al., 2016). All animal experiments in this study were monitored and handled according to an animal protocol approved by the Ethics Committee for Laboratory Animal of School of Life Science, Beijing Institute of Technology. The license number is SYXK (Beijing) 2012-2004.

#### Biochemistry analysis

Livers collected from the different groups were rinsed with PBS and cut into pieces, transferred to a homogenizer with physiological saline, smashed on an ice bath, and then centrifuged at 4,000 × g for 10 min. Supernatants were collected and stored at −80°C. The liver levels of the indices (Hyp, TNF-α) and serum levels of indices (ALT, AST, AKP, Alb, TBIL) were measured using a colorimetric analyser (Dri-Chem 3000, Japan) based on the manufacturer's instructions.

#### Liver histopathology

Liver tissues of each group were fixed in 10% neutral-buffered formalin, processed with routine histology procedures, and then embedded in paraffin and sliced into 5 μm sections. Subsequently, the samples were stained with haematoxylin-eosin and Masson's trichrome for histological assessment. Liver pseudolobule formation, collagen deposition, hepatocyte degeneration, and inflammatory infiltration were observed in the sections using a microscope. Immunohistochemstry (IHC) with α-SMA (at a 1:100 dilution in PBS) was applied to the specimens, as previously described (Zhou et al., 2014).

### Protein preparation, iTRAQ labeling, and analysis

#### Sample preparation

Control group, DMN model group and optimal effect group (H-ZYCH) were selected for proteomic analysis. Every liver tissue from control, DMN model and H-ZYCH rats was homogenized in SDS lysis buffer (1:5, w: v). The suspension was centrifuged at 12,000 × g for 15 min at 4°C, and the supernatant was collected (Eppendorf, Hamburg, Germany). The protein concentration of each sample was determined using the BCA protein assay kit. The control group was subsequently randomly divided into two subgroups: control-1 and control-2 (*n* = 5), the DMN model group was randomly divided into three subgroups: model-1, model-2 and model-3 (*n* = 3), and the H-ZYCH group was randomly divided into three subgroups: ZYCH-1, ZYCH-2 and ZYCH-3 (*n* = 3). Equal amounts of protein from each sample was mixed.

#### Protein digestion and iTRAQ labeling

A total of 200 μg proteins from each group was reduced with 10 mM DTT for 40 min at 50°C, and centrifuged with the addition of 200 μL UA. The sediment was mixed with 100 μL UA and 50 mM IAA (final concentration) for 1 h in the dark, centrifuged with 100 μL 0.5 M TEAB and collected again. After reduction and alkylation, the samples were digested with trypsin (Promega, WI, USA) at 37°C for 16 h, with a ratio of protein:trypsin of 50:1, and labeled using the iTRAQ Reagent-8 plex Multiplex Kit (Applied Biosystems, CA, USA) according to the manufacturer's protocol. The control subgroups (control-1 and control-2) were labeled with 113 and 114, the model subgroups (model-1, model-2, and model-3) were labeled with 115, 116, and 117 and the H-ZYCH subgroups (ZYCH-1, ZYCH-2, and ZYCH-3) were labeled with 118, 119, and 121.

#### High pH reversed-phase (RP) chromatography fractionation

The iTRAQ-labeled peptide mixtures were redissolved with 0.1% FA, and the supernatant was directly injected for HPLC after centrifugation. In the binary solvent system, mobile phase A contained 10 mM ammonium formate in water (pH 10.0), whereas mobile phase B contained 10 mM ammonium formate and 90% ACN (v/v, pH 10.0). LC separation was performed at a flow rate of 500 μL/min using a linear 40-min gradient consisting of 0%-5% B over 3 min, 5% B over 5 min, 5%-40% B over 28 min, 40–90% B over 2 min, and 90% B over 2 min. The fractions were collected starting at 8 min and ending at 38 min, with collection obtained every 1 min in a numbered tube. Finally, 10 fractions were mixed according to the tubes numbered mixed 1, 11, 21 tubes for a fraction; 2, 12, 22 tubes for a fraction; followed. Ten fractions were vacuum-dried for the following experiment.

#### LC–MS/MS analysis

Each fraction was desalted using a C18 Tip (Merck Millipore, Ds, Germany) with 50% ACN for the pre-balance and mixed solution (0.1% TFA, 5% ACN) for cleaning, followed by elution with 100 μL mixed solution (50% ACN, 0.1% TFA). Fractionated peptides were analyzed using an LTQ orbitrap ETD equipped with Easy-nLC 1000 (Thermo Fisher, MA, USA). The mobile phase A contained 0.1% formic acid (v/v) in water, whereas ACN was used as mobile phase B. Peptides were separated using a C18 column (1.8 μm, 0.15 × 1.00 mm) at an eluent flow rate of 300 nL/min using a linear 198 min gradient consisting of 2–25% B over 182 min, 25–35% B over 4 min, 35–90% B over 2 min, and 90% B over 1 min. The mass spectrum sampling method was performed using a data-dependent model as described elsewhere (Liu et al., 2017a). In brief, the medium isolation width was m/z 1.0, and the mass ranges of the MS1 and MS2 scans were set at 350–1600 in high sensitivity mode (resolution = 30,000).

#### Bioinformatics analysis

The resulting MS/MS spectra were searched against the UniProtKB database (http://www.uniprot.org/. 29,978 entries downloaded March 17, 2017) with taxonomy limited to *Rattus norvegicus* using the MaxQuant computational platform (version 1.5.2.8). For protein identification and quantification, a peptide mass tolerance of 20 ppm was allowed for intact peptide masses and 0.05 Da for fragmented ions. All identified peptides had an ion score above the Mascot peptide identity threshold, and a protein was identified if at least one such unique peptide match was apparent for the protein. For protein-abundance ratios measured using iTRAQ, we set a 1.2-fold change as the threshold and a *P*-value below 0.05 to identify significant changes between the DMN model group and the H-ZYCH group. Functional classifications and pathway annotations of the altered proteins were performed using the DAVID functional annotation tool (database for annotation, visualization, and integrated discovery, http://david.abcc.ncifcrf.gov/summary.jsp). Hierarchical clustering of the differentially expressed proteins used in the hcluster analysis were based on R Language.

### ELISA analysis

α-smooth muscle actin(α-SMA), collagen type IV (COLIV), superoxide dismutase (SOD), and malondialdehyde (MDA) were respectively measured with liver homogenate using the assay kit.

### Network pharmacology analysis

50 chemical compounds in ZYCH were obtained by separation (25 compounds) and the published literature (25 compounds deducting 4 repeated compounds). The three-dimensional (3D) geometric coordinates of 106 protein crystal structures of 282 altered proteins in proteomics were found and downloaded from the Protein Database Bank (PDB, http://www.rcsb.org/pdb/home/home.do), and these 106 proteins were identified as protein targets. Subsequently, molecular docking calculations were performed for 50 chemical compounds and 106 proteins targets using the Libdock protocol under the protein-ligand interaction section in Discovery Studio® 3.1 (Accelrys, San Diego, USA), which allowed the ligand to be separately and structurally rearranged in response to the receptor. Docking was carried out as described elsewhere (Chen et al., 2017). The top 20 percent of the compounds interacting with each protein were selected according to the libdock score. Protein targets and selected compounds were uploaded to Cytoscape software (vision 3.4.0). A set of networks was generated, in which nodes with red or green colors represented up or down-regulated proteins. The biological relationship between two nodes was expressed as an edge (line). In addition, the networks were ranked by compound types to represent the significance of each molecule type.

### Cell experiments

#### Cell culture

Rat hepatic stellate cells-T6 (HSCs-T6) were purchased from the cancer hospital, Chinese Academy of Medical Sciences (Beijing, China), and cultured in RPMI 1640 medium (GIBCO, Gaithersburg, MD, USA) supplemented with 10% fetal bovine serum (FBS, Tianhang Biotechnology, Zhejiang, China) and 1% penicillin-streptomycin (PS) at 37°C in a humidified atmosphere with 5% CO_2_.

#### HSCs-T6 viability assay

HSCs-T6 were plated into a 96-well cell culture plate at a density of 8 × 10^3^ cells/well and incubated at 37°C for 24 h in a 5% CO_2_ incubator. Subsequently, the cells were washed twice with PBS, followed by incubation with compounds 1-25 (200, 300, 400, 500, 600 μM) for up to 24 h at 37°C in a 5% CO_2_ incubator. Next, 20 μL of MTS reagent (Promega, WI, USA) was added to each well, and the cells were incubated for another 3 h. Finally, the absorbance of each well was measured at 492 nm with a Labsystems Multiskan MS microplate reader (Thermo Electron, Massachusetts, USA).

#### Stimulated HSCs-T6 inhibition assay

HSCs-T6 were seeded into a 96-well cell culture plate at a density of 8 × 10^3^ cells/well and incubated at 37°C for 24 h in a 5% CO_2_ incubator. Subsequently, the cells were washed twice with PBS and serum starved overnight, followed by incubation with compounds 1-25 (1, 25, 50, 75, 100 μM) accompanied by stimulation with 10 ng/mL TGF-β1 for up to 24 h at 37°C in a 5% CO_2_ incubator. Next, 20 μL MTS reagent (Promega, WI, USA) was added to each well, and the cells were incubated for another 3 h. Finally, the absorbance of each well was measured at 492 nm.

### Statistical analysis

The results are expressed as the means ± standard deviation. The significance values were calculated using SPSS 17.0 software. Data were analyzed by homogeneity of variance and one-way analysis of variance (ANOVA). *P*-values < 0.05 were considered statistically significant.

## Result and discussion

### ZYCH compound data preparation

Repeated column chromatography of the ZYCH extract resulted in the isolation of 25 known compounds (Compounds **1**-**25**, Supplementary Figure 1). The compounds were identified as chinensin (**1**) (Lopez et al., 1996), (–)-matairesinol (**2**) (Umezawa et al., 1991), octacosanoic acid (**3**) (Chen et al., 2013), diphyllin (**4**) (Annapoorani et al., 1984), kaerophyllin (**5**) (Mikaya et al., 1981), α-peltatin (**6**) (Raffauf et al., 1987), butyrolactone (**7**) (Li et al., 2006), demethylyatein (**8**) (Sanfeliciano et al., 1991), bupleurumin (**9**) (Kim and Yun-Choi, 2007), suchilactone (**10**) (Banerji et al., 1984), matairesinol monoglucoside (**11**) (Sakamura et al., 1980), indigoticalignanoside A (**12**) (Zhang et al., 2013), (–)-matairesinol 4-O-glucoside (**13**) (Yang et al., 2015), vanillic acid 4-β-D-glucoside (**14**) (Nugraha et al., 2015), wenchuanensin (**15**) (Luo et al., 1993), kaempferol 3-O-neohesperidoside (**16**) (Budzianowski et al., 1985), asicariside b1 (**17**) (Sasaki et al., 2013), citroside A(**18**) (Cho et al., 2014), dehydrodiconiferyl alcohol 4-O-β-D-glucopyranoside (**19**) (Tan et al., 2005), saikosaponin B_2_ (**20**) (Ishii et al., 1980), styraxlignolide C (**21**) (Di et al., 2013), 11α-methoxy saikosaponin F (**22**) (Ding et al., 1986), saikosaponin H (**23**) (Shimizu et al., 1985), clinoposaponin XI (**24**) (Mori et al., 1994), saikosaponin C (**25**) (Ishii et al., 1980). The above compounds were isolated from this plant for the first time except compounds **1**, **3**, **20**, and **25**. Twenty-five reported compounds of ZYCH were obtained from the literature (deducting 4 repeated compounds): 4 saikosaponins, 5 flavonoids, 6 sterols, 5 lignans, 3 ketolides, and 2 sugar alcohols (Supplementary Figure 2). Fifty chemical ZYCH compounds were obtained in this study: 9 triterpenoid saponins, 6 flavonoids, 6 sterols, 20 lignans, 3 ketolides, 2 sugar alcohols, 2 sesquiterpenoids, 1 phenolic acid, and 1 long-chain aliphatic acid.

### Effects of ZYCH on liver fibrosis

**Figure 1:**
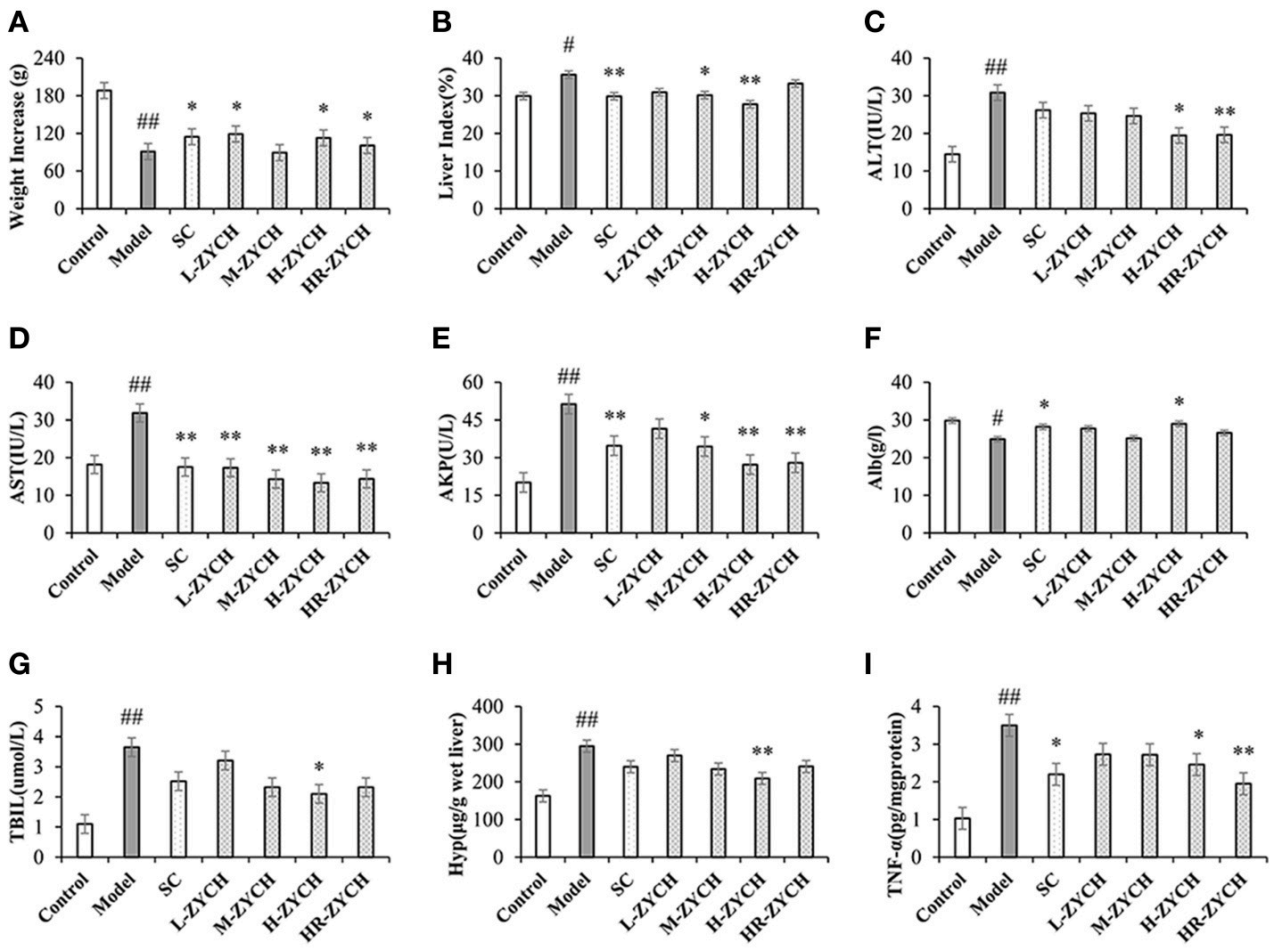
Body weight increase, liver index and biochemical assays results after ZYCH treatment in DMN-induced liver fibrosis. **(A,B)** body weight increase and liver index; **(C-G)** serum ALT, AST, AKP, Alb, TBIL level; **(H,I)** the production of Hyp and TNF-α in liver tissues. #*p* < 0.05, ##*p* < 0.01 vs. control group, ^*^p < 0.05, ^**^p < 0.01 vs. model group.

After initiation of the DMN injections, the liver fibrosis rat model was evaluated based on indicators of body weight increments and the liver index. Compared with the control group, DMN administration considerably reduced body weight and increased the liver index in rats, while ZYCH treatment had a significant effect on body weight and the liver index (Figure 1).

Analysis of serum functional enzymes, including ALT, AST and AKP, has vital clinical value for the diagnosis of liver function (Tabet et al., 2016). As shown in Figure 1, DMN model group was significantly increased serum ALT, AST, and AKP activities compared with the control group (*p* < 0.01). Conversely, ZYCH inhibited the DMN-induced serum ALT, AST, and AKP activities compared with the model group. In particular, H-ZYCH rats showed a significant decrease in the activities of ALT, AST, and AKP (*p* < 0.01, *p* < 0.05). Additionally, the serum levels of Alb and TBIL are important indicators that are closely related to liver function. H-ZYCH significantly increased Alb and decreased TBIL compared with the model group (*p* < 0.05) (Figure 1). The results also showed that H-ZYCH group could decreased Hyp and increased TNF-α in hepatic level (*p* < 0.01) (Figure 1). Compared with the positive SC group, the H-ZYCH group showed more effective results to maintain the normal levels of serum ALT, AST, AKP, TBIL and hepatic Hyp, TNF-α.

**Figure 2:**
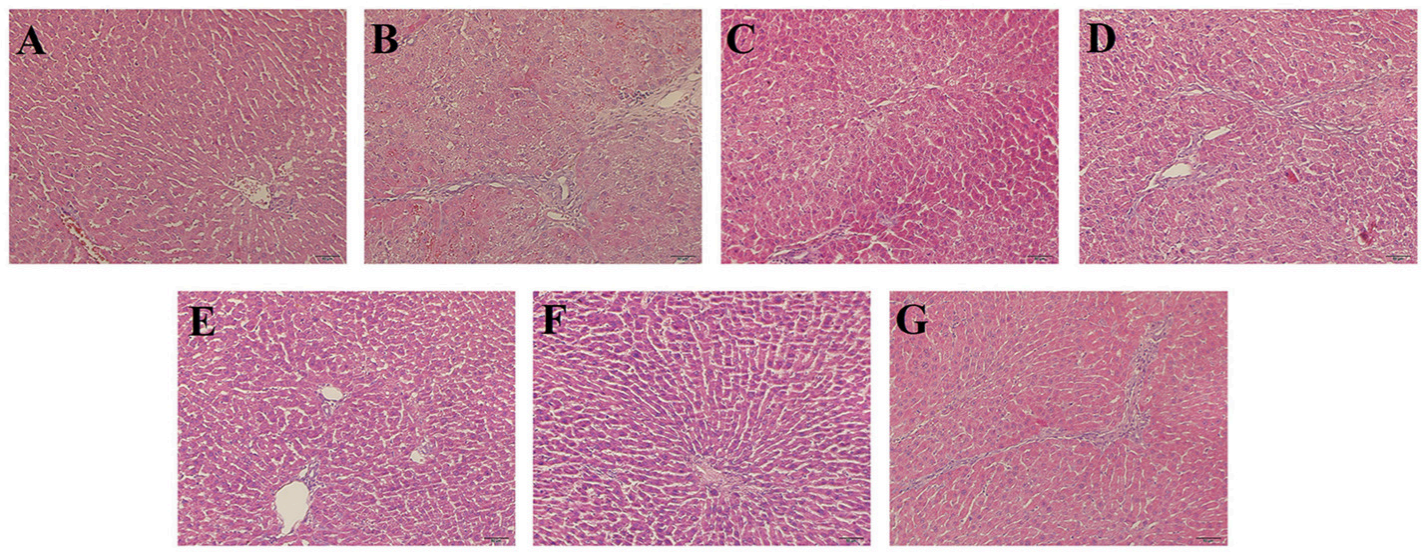
Effects of ZYCH on hepatic histopathological changes of DMN-induced liver injury rats. Hepar sections by hematoxylin-eosin (HE) staining at a magniication of ×100. **(A)**. control group, **(B)**. model group, **(C)**. SC group, **(D)**. L-ZYCH group, **(E)**. M-ZYCH group, **(F)**. H-ZYCH group, and **(G)**. HR-ZYCH group.

**Figure 3:**
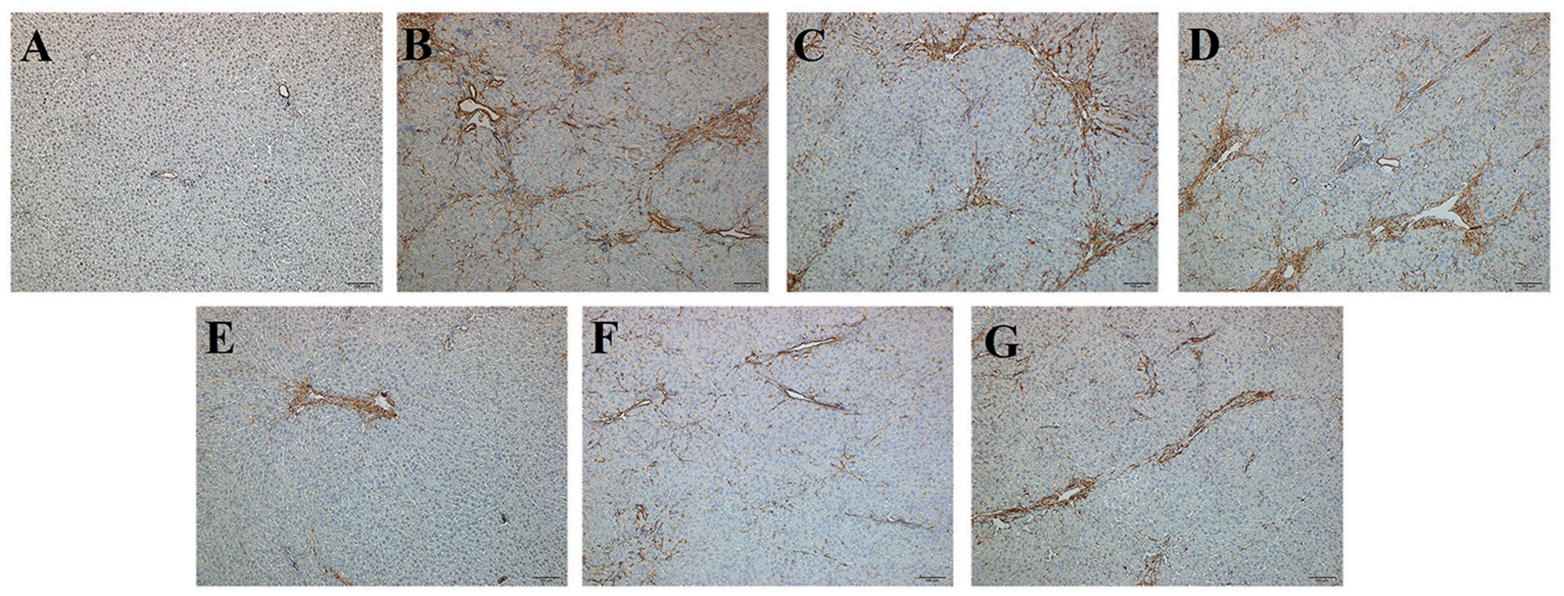
Effects of ZYCH on α-SMA IHC changes of DMN-induced liver injury rats. Original magnification: 100×. **(A)**. control group, **(B)**. model group, **(C)**. SC group, **(D)**. L-ZYCH group, **(E)**. M-ZYCH group, **(F)**. H-ZYCH group, and **(G)**. HR-ZYCH group.

To evaluate histological changes in the liver, H&E, Masson's Trichrome and α-SMA IHC-stained tissue sections from each group were assessed. In H&E-stained liver tissue of the DMN-treated groups exhibited more fibrosis, necrotic hepatocytes, and degenerative hepatocytes than the control group (Figures 2A,B). The groups treated with SC, high-dose and higher-dose ZYCH correspondingly appeared to exhibit an alleviation of the pathological injuries (Figures 2C–G). Masson's staining showed collagen fibril accumulation, pseudolobuli and tubercle formation in the DMN model group (Supplementary Figure 3). The rat liver morphology also appeared to be normal in the SC and H-ZYCH group. α-SMA IHC staining revealed a trend similar to that observed in H&E and Masson's Trichrome staining (Figure 3), further suggesting that the liver histology results were consistent with the liver function test.

### iTRAQ analysis of differentially expressed proteins

**Figure 4:**
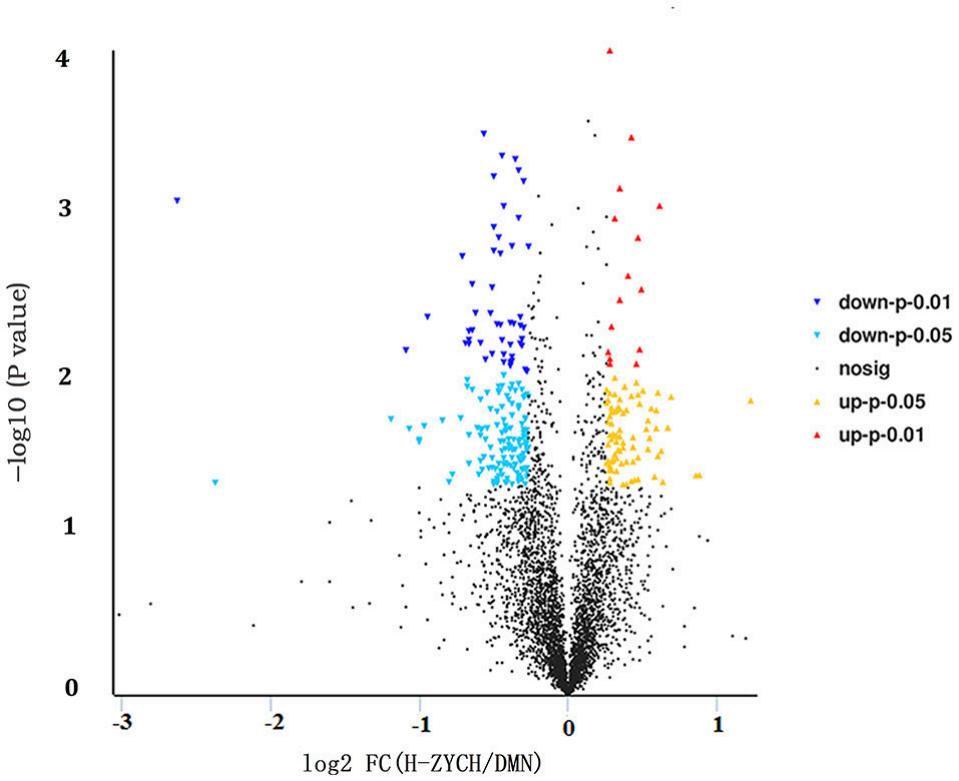
Volcano plot analysis of differentially expressed proteins between H-ZYCH and DMN-model groups. Volcano plot of ratios and *p*-value represented the protein abundance changes in the comparison between H-ZYCH and DMN-model groups. Proteins with *p* < 0.05 and above/below 1.2-fold changes are identified as proteins with significant changes. Down-regulated with *p* < 0.01 (dark blue dots), down-regulated with 0.01 ≤ *p* < 0.05 (light blue dots), up-regulated with *p* < 0.01 (red dots), and up-regulated with 0.01 ≤ *p* < 0.05 (yellow dots).

To investigate the molecular mechanism of ZYCH on DMN-induced liver fibrosis, iTRAQ technology in combination with LC-MS/MS was applied to detect differentially expressed proteins in liver samples from DMN model rats and H-ZYCH rats. A total of 4155 distinct proteins were identified and quantified using MaxQuant computational platform (version 1.5.2.8) against the UniProtKB database (http://www.uniprot.org/) with taxonomy limited to *R. norvegicus*, whereas 282 proteins were altered in H-ZYCH group compared with the DMN model group (fold changes > 1.2 or < 0.83, *p* < 0.05 as the threshold), 185 up-regulated proteins and 97 down-regulated proteins (Figure 4). Hierarchical clustering was generated to show the differentially proteins the two groups (Supplementary Figure 4).

**Figure 5:**
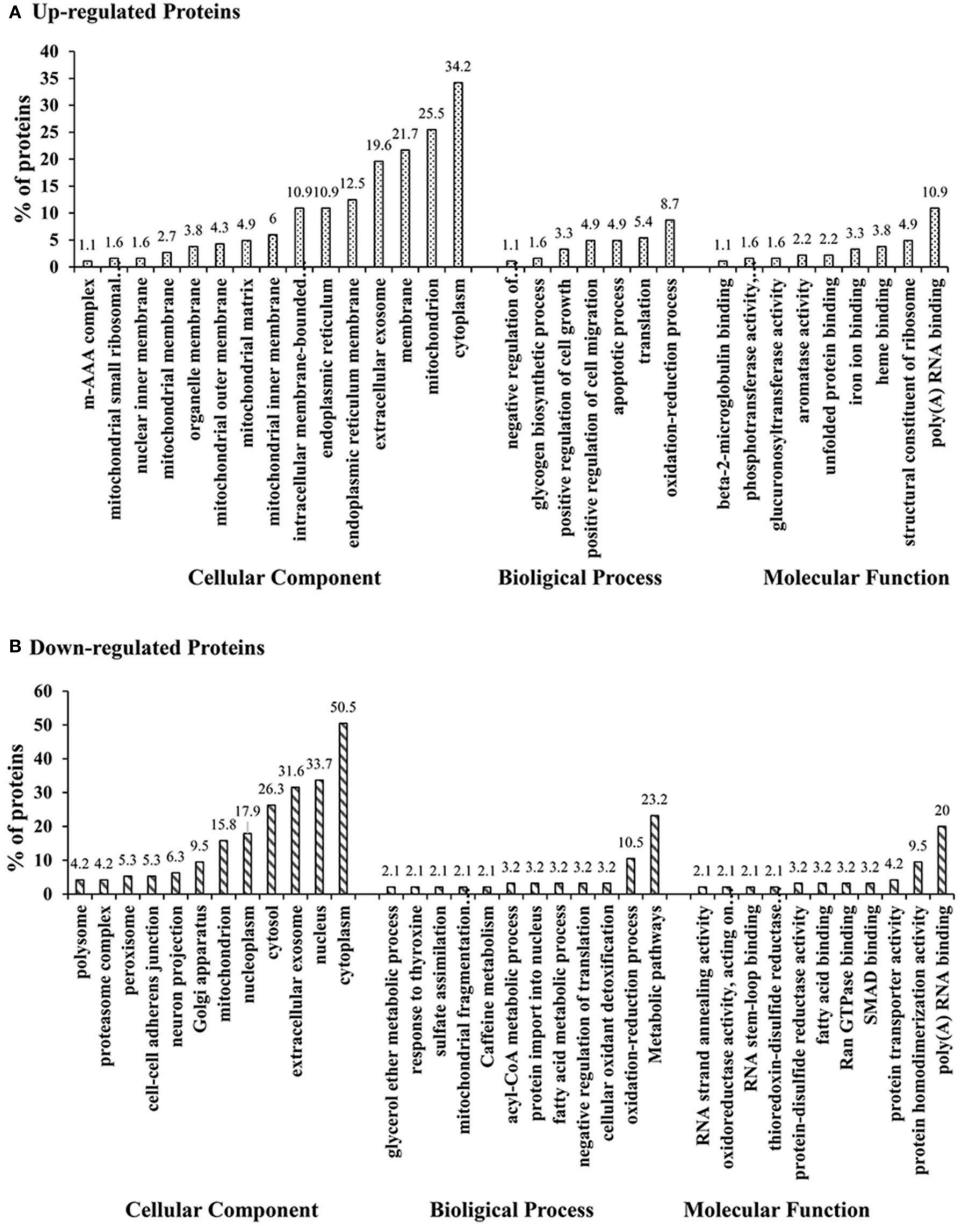
GO classification of up-regulated altered proteins and down-regulated altered proteins between H-ZYCH and DMN-model groups. **(A)**. GO classification of up-regulated proteins; **(B)**. GO classification of down-regulated proteins.

The differentially expressed proteins in GO were analyzed using DAVID. the GO annotation of cellular component (CC), molecular function (MF), and biological process (BP) of all identified proteins, all altered proteins, up-regulated proteins, and down-regulated proteins were annotated separately (Supplementary Figures 5–7). In the CC annotation, the percentage of proteins assigned to extracellular exosome, cytosol, and nucleoplasm in all altered proteins appeared to be decreased compared with all identified proteins (Supplementary Figure 5). Up-regulated proteins assigned to the mitochondrion, cytosol, nucleoplasm, endoplasmic reticulum membrane, and intracellular membrane-bound organelles, down-regulated proteins assigned to the cytoplasm, mitochondrion, membrane, endoplasmic reticulum membrane, and intracellular membrane-bound organelles. The CC annotation of up/down-regulated proteins were all clearly altered compared with that of all altered proteins (Figure 5). In the MF annotation, compared with all identified proteins, the percentage of the altered proteins increased in protein kinase binding but decreased in protein binding and ATP binding (Supplementary Figure 6). The percentage of up-regulated proteins assigned to structural constituents of the ribosome and down-regulated proteins assigned to protein homodimerization activity were increased compared with all altered proteins in the BP annotation, the clustered BP annotation of up-regulated and down-regulated proteins were entirely different (Supplementary Figure 7). Additionally, based on GO annotation analysis, clusters of drug metabolism, oxidative stress, biomolecular synthesis and metabolism, positive regulation of cell growth, extracellular matrix deposition, and focal adhesion were significantly regulated in H-ZYCH treatment rats in comparison to DMN model rats.

#### Drug metabolism

The liver is the most important organ for drug metabolism. We found that UDP-glucuronosyltransferase (UGT2A3), UDP-glucuronosyltransferase (UGT2B35), UDP glucuronosyltransferase family 1 member A1 (UGT1A1), protoporphyrinogen oxidase (PPOX), focal adhesion kinase 1 (FADK1), long-chain-fatty-acid–CoA ligase 1 (LACS1), and 4-aminobutyrate aminotransferase, mitochondrial (ABAT) were up-regulated. Adenylate kinase isoenzyme 1(AK1), thioredoxin (TRX), and glutathione S-transferase (GSTM4), which are involved in drug metabolism, were decreased (Table 1).

**Table 1:** List of the altered proteins of DMN-model and H-ZYCH rats.

**Keywords**	**Protein ID**	**Protein names**	**Fold change**	**Number of targeted compounds**
Drug metabolism	A0A0G2JW02_RAT	UDP-glucuronosyltransferase 2A3	1.61	—
	A0A0G2K727_RAT	UDP-glucuronosyltransferase 2B35	1.21	8
	A0A0G2K7Q6_RAT	Adenylate kinase isoenzyme 1	0.79	9
	D3ZVN7_RAT	Protoporphyrinogen oxidase	1.25	7
	FAK1_RAT	Focal adhesion kinase 1	1.35	5
	THIO_RAT	Thioredoxin	0.66	9
	ACSL1_RAT	Long-chain-fatty-acid–CoA ligase 1	1.33	1
	GABT_RAT	4-aminobutyrate aminotransferase, mitochondrial	1.27	1
	Q5BK56_RAT	Glutathione S-transferase	0.75	7
	UD11_RAT	UDP-glucuronosyltransferase 1-1	1.37	6
Oxidative stress	B0BNF9_RAT	Hydroxyacid oxidase 1	0.78	2
	F1LNW3_RAT	Acyl-coenzyme A oxidase	0.83	6
	THIO_RAT	Thioredoxin	0.66	9
	TXNL1_RAT	Thioredoxin-like protein 1	0.79	—
Biomolecular synthesis and metabolism–Carbohydrate	A0A096MJY6_RAT	1,4-alpha-glucan-branching enzyme 1	2.27	2
	A0A0G2JXP1_RAT	Glycogenin-1	1.20	9
	AKT1_RAT	RAC-alpha serine/threonine-protein kinase	1.39	6
	ENOA_RAT	Alpha-enolase	0.82	1
Biomolecular synthesis and metabolism–fat	F1M1W1_RAT	Acyl-CoA synthetase medium-chain family member 1	1.28	9
	FAS_RAT	Fatty acid synthase	0.72	5
	ECH1_RAT	Delta(3,5)-Delta(2,4)-dienoyl-CoA isomerase	1.22	1
	CBR4_RAT	Carbonyl reductase family member 4	1.22	6
Biomolecular synthesis and metabolism–protein	D4A904_RAT	N-acetylglutamate synthase	1.20	5
	Q5XFW4_RAT	Mitochondrial ribosomal protein L13	1.41	—
	D4A4A9_RAT	Mitochondrial ribosomal protein L19	1.43	—
	B2RYT4_RAT	Mitochondrial ribosomal protein S14	1.39	—
	D4A7X1_RAT	Mitochondrial ribosomal protein S16	1.52	—
	RT07_RAT	28S ribosomal protein S7, mitochondrial	1.30	—
	RL12_RAT	60S ribosomal protein L12	1.56	—
	RL32_RAT	60S ribosomal protein L32	1.20	—
Positive regulation of cell growth	A0A0G2K562_RAT	Disintegrin and metalloproteinase domain-containing protein 10	1.25	4
	FAK1_RAT	Focal adhesion kinase 1	1.35	5
	AKT1_RAT	RAC-alpha serine/threonine-protein kinase	1.39	6
	SUV3_RAT	ATP-dependent RNA helicase, mitochondrial	1.32	2
Extracellular matrix deposition and focal adhesion	A0A0G2JVZ6_RAT	Integrin subunit alpha V	1.23	—
	A0A0G2JWJ0_RAT	Protein phosphatase 1 regulatory subunit	0.78	—
	A0A0G2KAN1_RAT	Collagen alpha-2(I) chain	1.59	—
	SYNP2_RAT	Synaptopodin-2	1.37	—
	D4A7U1_RAT	Zyxin	0.73	—
	F1LQ00_RAT	Collagen type V alpha 2 chain	2.13	—
	F1M6H4_RAT	Collagen, type VI, alpha 4	5.26	—
	CO1A1_RAT	Collagen alpha-1(I) chain	1.49	—

UGT2A3, UGT2B35, and UGT1A1 belong to UDP glucuronosyltransferase family (UGTs) (Tanemura et al., 2017). UGTs are the main phase II drug metabolic enzymes that can catalyze glucuronic acid from UDP-glucuronic acid (UDPGA) to transfer to exogenous substances, and improve water solubility of the receptor, then expel from the body. Previous report has indicated that UGT1A1 can be used as the biomarker of HCV treatment (Costa et al., 2011), which has been confirmed to increase along with the liver fibrosis progress of HCV patients (Sumida et al., 2000), It has been demonstrated in early studies that TRX increase with the progression of hepatic fibrosis in HCV-infected patients, and patients with higher levels of TRX have demonstrated anti-interferon therapy effect. TRX is decreased in H-ZYCH group compared with model group, which is in accordance with the early report (Sumida et al., 2000).

#### Oxidative stress

Alterations of oxidoreductases were observed, implying a reduction of oxidative stress, which including the down-regulation of the proteins acyl-CoA oxidase 2 (ACOX2), hydroxyacid oxidase 1 (HAO1), thioredoxin 1 (TXN1), and thioredoxin-like 1 (TXNL1) in H-ZYCH rats (Table 1). ACOX2, as acyl-CoA oxidase, can catalyze acyl-CoA and O_2_ to generate and H_2_O_2_, then H_2_O_2_ will aggravate the lesion degree of oxidative stress (Vilarinho et al., 2016). To further confirm whether ZYCH could reduce oxidative stress in rats, the contents of MDA and SOD activity were measured. The H-ZYCH group showed a significant reduction of SOD activity and an increase in MDA content in comparison to the DMN model group (*p* < 0.05) (Supplementary Figures 8A,B). The result suggested that after ZYCH treatment, the DMN-induced oxidative injury of liver tissue was reduced.

#### Biomolecular synthesis and metabolism

1,4-alpha-glucan branching enzyme 1 (GBE1), AKT serine/threonine kinase 1 (AKT1), and glycogenin 1 (GYG1), which are involved in the glycogen biosynthetic process, were increased. Conversely, alpha-enolase (ENO1) (Peng et al., 2013), as the key enzyme in glycolysis, was decreased. The altered proteins in glycometabolism indicated that after ZYCH treatment, abnormal glycogen decomposition, and consumption of liver caused by DMN has been alleviated, and glycogen synthesis has been recovered for energy storage. Proteins assigned to fatty acid metabolism, such as acyl-CoA synthetase medium-chain family member 1 (ACSM1), carbonyl reductase family member 4 (CBR4), and enoyl-CoA hydratase 1 (ECH1) were increased. Fatty acid synthase (Fasn) (Mullen and Yet, 2015) as an important enzyme in the fatty acid biosynthetic process was decreased (Table 1). The results suggested that in H-ZYCH group, fatty acid synthesis was retarded and fatty acid metabolism was accelerated. In addition, proteins involved in amino acids biosynthesis were also altered, N-acetylglutamate synthase (NAGS) (Dercksen et al., 2016) as an enzyme participating in the arginine biosynthetic process, and 7 proteins involved in ribosomal structural constituents were all up-regulated, which indicated that amino acids synthesis was enhanced in ZYCH-treated rats. Based on the above results, we considered that after ZYCH treated, the metabolic disorder of biological macromolecules caused by DMN tended to be stable. The excessive accumulation of fatty acids accelerated consumption, and the synthesis of glycogen and protein were recovered.

#### Positive regulation of cell growth

Considering that liver fibrosis induced by DMN is a process of hepatocyte necrosis and apoptosis, we found, as expected, that proteins involved in cell growth were increased after ZYCH treatment (Table 1), including disintegrin, metalloproteinase domain-containing protein 10 (Adam10), AKT serine/threonine kinase 1 (AKT1) (Fan et al., 2015), ATP-dependent RNA helicase SUPV3L1, mitochondrial (SUPV3L1), and focal adhesion kinase 1 (PTK2) (Heinhuis et al., 2012; Zhao et al., 2017).

#### Extracellular matrix deposition and focal adhesion

Focal adhesions are large, dynamic protein complexes through which the cytoskeleton of a cell connects to the ECM (Kumar et al., 2014). We found that 2 proteins involved in focal adhesion, protein phosphatase 1 regulatory subunit 12A (PPP1R12A) and zyxin (ZYX), were decreased (Table 1). However, some proteins assigned to the ECM were increased, such as integrin subunit alpha V (ITGAV), collagen alpha-2 (I) chain (COL1a2), collagen type V alpha 2 chain (COL5a2) and collagen, type VI, alpha 4 (COL6a4), collagen alpha-1 (I) chain (COL1a1). The exact biological significance of these proteins must be further investigated. In order to detect liver ECM deposition degree, we selected α-SMA and COLIV for further confirmation by ELISA and IHC staining. The reduced α-SMA expression in H-ZYCH group was determined by ELISA (*p* < 0.05) (Supplementary Figure 8C). IHC staining revealed that the expression of α-SMA was prominent in DMN-model livers and decreased in H-ZYCH rats (Figure 3). COLIV was confirmed by ELISA and showed a significant increase in the DMN model group (*p* < 0.01) and decrease in the H-ZYCH group (*p* < 0.01) (Supplementary Figure 8D). The result indicated that DMN-induced ECM deposition had been obviously alleviated in H-ZYCH group.

### Results of the network pharmacology-based analysis

To understand the complex interaction of compounds and their corresponding targets at a system level, compound-target network (C-T network) was established. the docking analysis was performed between 50 ZYCH chemical compounds and 106 potential liver fibrosis targets (60 up-regulated proteins, 46 down-regulated proteins). Top 20 percent of the docked compounds of each target were obtained, then the docking results were summarized to construct the C-T network (Supplementary Table 2). The C-T network embodied 218 nodes and 831 edges, among which 121 nodes and 542 edges belong to the compound-protein interactions, and these 121 nodes include 91 target protein nodes and 30 compound nodes. The remaining nodes and edges belong to protein-protein interactions. According to the statistical results of the network analysis plugin module, the mean degree value (the number of associated targets) of the compounds were 18, indicating that most of the compounds regulated multiple targets to exert various therapeutic effects. The mean compounds of each target were 6, showing that most of the targets can interact with multiple compounds simultaneously. The result reflected the TCM characteristics of multi-targets and multi-compounds.

**Figure 6:**
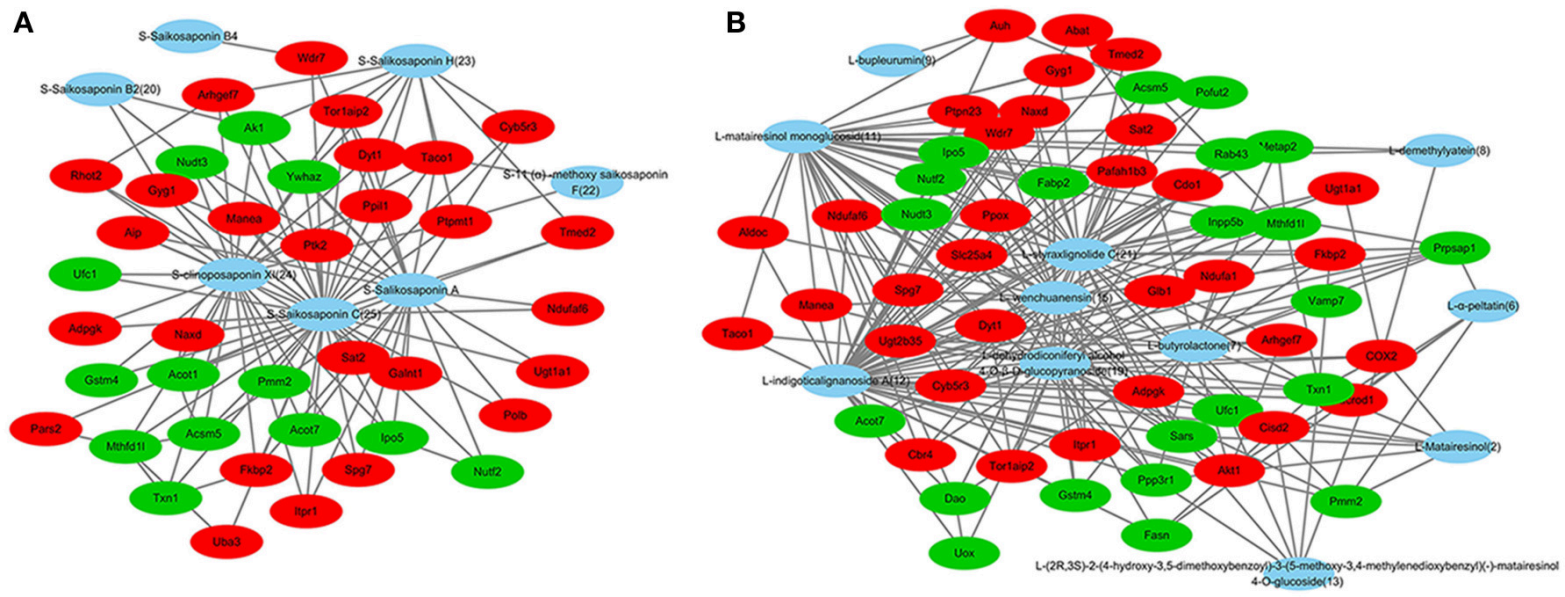
Compound–target networks related to ZYCH effects on DMN-induced liver fibrosis. **(A)** C-T network of triterpenoid saponins and targets. **(B)** C-T network of lignans and targets. Red ellipses are up-regulated proteins, and green ellipses are down-regulated proteins in proteomics. Blue ellipses are compounds.

C-T networks based on each type of compounds were established. The network of triterpenoid saponins and their targets embodied 46 nodes and 113 interactions (Figure 6A). 46 nodes include 7 compounds and 39 target proteins. These 7 lead compounds out of 9 triterpenoid saponins indicated that the triterpenoid saponins had a strong binding effect to the target protein in the molecule docking. Specifically, compd.**25**, **24** and saikosaponin A, respectively acting on 39, 25 and 24 targets, are key compounds in this network. The network of lignans and their targets embodied 66 nodes and 237 interactions (Figure 6B). 66 nodes include 11 compounds and 55 target proteins. These 11 lead compounds out of 20 lignans indicated that the lignans had a strong binding effect with the target protein in the molecule docking. Compd. **12**, **21**, **11**, **19**, **15** respectively acting on 55, 37, 35, 30, and 26 targets, are at important positions in this C-T network. The network of sterols and targets embodied 26 nodes and 24 interactions. 26 nodes include 4 compounds and 22 target proteins. Among them, daucosterin can be linked to 17 target proteins and at an important position in the C-T network, while cholest-7-en-3β-ol, stigmasta-7, 25-dien-3-ol, and 7-sitoster-3β-ol are associated with 2 targets, respectively. The results indicated that triterpenoid saponins and lignans maybe the crucial active compounds in view of their multi-targets effects and important roles in the C-T networks.

C-T networks also showed that key targets were recognized by multiple compounds. UGT2B35, AK1 and TXN1, which participate in drug metabolism, were targeted by 8, 9, and 9 compounds. ACOX2 involved in oxidative stress was targeted by 6 compounds. GYG1 and AKT1, which involved in the glycogen biosynthetic process, were targeted by 9 and 6 compounds. ACSM1 and CBR4 involved in fatty acid metabolism, were targeted by 9 and 6 compounds. Most of these compounds were triterpenoid saponins and lignans, especially the compounds targeting at UGT2B35 and AKT1. 2 triterpenoid saponins and 6 lignans were targeted to TXN1. 4 triterpenoid saponins and 3 lignans were targeted to GYG1. AKT1targeted compounds were mostly lignans except for 1 long-chain fatty acids. 6 compounds targeted with UGT2B35 were lignans (Table 1).

### Effects of ZYCH compounds on HSCs-T6

Firstly, the effect of ZYCH compounds **1**-**25** on the viability of HSCs-T6 was evaluated using the MTS assay, which demonstrated that most ZYCH compounds (<100 μM) had no significant effect on the viability of HSCs-T6 at 24 h (Table 2). Secondly, the inhibitory effect of ZYCH compounds **1**-**25** on TGF-β1-stimulated HSCs-T6 was evaluated. As shown in Table 2, compd. **25** demonstrated the greatest inhibitory activity toward TGF-β1-stimulated HSCs-T6 among compounds **1**-**25**, which suggested that compd. **25** (34.48 ± 0.57 μM) might possess the most potent activity for ZYCH anti-liver fibrosis. The docking and C-T network analysis predicted that triterpenoid saponins (compd. **24**, **25**) and lignans (compd. **2**, **21**, **11**, **19**, **15**) might be the main active ingredients of ZYCH on liver fibrosis. The IC50 value of these compounds on stimulated HSCs-T6 were investigated and showed strong inhibitory activity than the others. The results were basically consistent with the prediction of the C-T network.

**Table 2:** Effects of compounds **1**-**25** from ZYCH on HSC-T6 cells($\bar{x}$ ± s).

**Types**	**Compounds**	**Viability of HSCs-T6 IC50(uM)**	**Inhibition of stimulated HSCs-T6 IC50(uM)**
Lignans	1	298.92 ± 9.37	–
	2	277.49 ± 22.34	–
	4	354.32 ± 21.01	107.98 ± 9.64
	5	276.81 ± 18.34	109.70 ± 7.89
	6	330.04 ± 18.91	92.12 ± 1.32
	7	312.92 ± 13.26	83.96 ± 4.63
	8	296.30 ± 24.06	–
	9	232.87 ± 6.63	–
	10	315.12 ± 14.82	133.76 ± 3.28
	11	426.97 ± 34.81	65.55 ± 0.92
	12	422.20 ± 26.62	91.30 ± 2.57
	13	596.30 ± 37.86	–
	15	670.75 ± 39.46	89.22 ± 4.25
	19	433.78 ± 21.45	93.12 ± 8.33
	21	389.48 ± 11.41	84.15 ± 2.78
Triterpenoid saponins	20	294.56 ± 27.96	83.54 ± 2.74
	22	387.70 ± 31.75	–
	23	577.97 ± 27.48	130.49 ± 4.84
	24	–	86.87 ± 8.52
	25	164.18 ± 6.12	34.48 ± 0.57
Fatty acids	3	362.30 ± 17.42	–
Phenolic acids	14	1097.65 ± 46.32	148.57 ± 7.72
Flavonoids	16	818.71 ± 19.89	89.35 ± 3.94
Sesquiterpenoids	17	–	–
	18	–	107.47 ± 4.82

## Conclusions

In this study, 25 known compounds were separated from ZYCH, among which 21 compounds were first isolated from ZYCH. The effects of ZYCH on DMN-induced liver fibrosis in rats were assessed, and H-ZYCH group showed optimal effect, which was selected for further proteomic analysis. Two hundred and eighty-two altered proteins were found in H-ZYCH group in comparison to DMN model group. Based on the GO annotation, clusters of drug metabolism, oxidative stress, biomolecular synthesis and metabolism, positive regulation of cell growth, extracellular matrix deposition, and focal adhesion were significantly regulated. The interaction of ZYCH compounds and anti-liver fibrosis targets were analyzed by molecular docking. Subsequently, a set of C-T networks were generated by Cytoscape. The results indicated that triterpenoid saponins and lignans were key compounds in the C-T networks for they had high libdock scores and the most targets. Then the inhibitory effect of ZYCH compounds **1**-**25** on TGF-β1-stimulated HSCs-T6 was evaluated, triterpenoid saponins (compd. **24**, **25**) and lignans (compd. **2**, **21**, **11**, **19**, **15**) had strong inhibitory activity. These findings suggested that triterpenoid saponins and lignans might be the major active compounds in ZYCH anti-liver fibrosis and target at proteins involved in drug metabolism, oxidative stress, biomolecular synthesis and metabolism, positive regulation of cell growth, and focal adhesion. The exact biological significance of these ZYCH compounds need be further investigated. The combination of network pharmacology with proteomic analysis may provide a forceful tool for exploring the effect mechanism of TCM and identifying bioactive ingredients and their targets.

## Author contributions

XL carried out most of the studies, performed statistical analysis, and wrote the manuscript. KL and YG participated in the animal experiments. YH and YS participated in the data processing work. WM provided professional advices. RD and YD designed the study and revised the manuscript. All authors have read and approved the final version.

### Conflict of interest statement

The authors declare that the research was conducted in the absence of any commercial or financial relationships that could be construed as a potential conflict of interest.

## Supplementary material

Supplementary Figure 1 Structures of compounds **1-25** from ZYCH.

Supplementary Figure 2 Structures of the reported compounds from ZYCH in literatures.

Supplementary Figure 3 Effects of ZYCH on Masson's trichrome staining changes of DMN-induced liver injury rats. Original magnification: 100×. **(A)** Control group, **(B)** model group, **(C)** SC group, **(D)** L-ZYCH group, **(E)** M-ZYCH group, **(F)** H-ZYCH group, and **(G)** HR-ZYCH group.

Supplementary Figure 4 Hierarchical cluster analysis of differentially expressed proteins between H-ZYCH and DMN-model groups. The rows represent each protein and the column means different groups. The color in the graph indicates the relative expression of the protein in the sample, red represents the higher expression level and the green represents the lower expression. The left independent column represents the classification of the protein (10 classes).

Supplementary Figure 5 GO classification of all identified proteins, all altered proteins, up-regulated altered proteins and down-regulated altered proteins between H-ZYCH and DMN-model groups in cellular component. Union of The first 15 subtypes of all identified proteins and all altered proteins in GO classification were analyzed.

Supplementary Figure 6 GO classification of all identified proteins, all altered proteins, up-regulated altered proteins and down-regulated altered proteins between H-ZYCH and DMN-model groups in molecular function. Union of The first 15 subtypes of all identified proteins and all altered proteins in GO classification were analyzed.

Supplementary Figure 7 GO classification of all identified proteins, all altered proteins, up-regulated altered p6roteins and down-regulated altered proteins between H-ZYCH and DMN-model groups in biological process. Union of The first 15 subtypes of all identified proteins and all altered proteins in GO classification were analyzed.

Supplementary Figure 8 ELISA analysis for proteomics validation of H-ZYCH and DMN-model rats in liver tissues. **(A)** The activity of SOD; **(B)** the production of MDA; **(C)** the production of α-SMA; **(D)** the production of COLIV. ^#^*p* < 0.05, ^*##*^*p* < 0.01 vs. control group, ^*^*p* < 0.05, ^**^*p* < 0.01 vs. model group.

Supplementary Table 1 The experimental schedule of ZYCH anti-liver fibrosis induced by DMN.

Supplementary Table 2 The molecular docking results of ZYCH anti-liver fibrosis protein targets.

## References

[B1] Annapoorani K. S. Damodaran C. Sekharan P. C. (1984). Spectrofluorodensitometric determination of diphyllin, a cystostatic lignan isolated from *Cleistanthus collinus*. Pharmazie 39, 716–717. 6522456

[B2] Ashour M. L. El-Readi M. Youns M. Mulyaningsih S. Sporer F. Efferth T.. (2009). Chemical composition and biological activity of the essential oil obtained from *Bupleurum marginatum* (*Apiaceae*). J. Pharm. Pharmacol. 61, 1079–1087. 10.1211/jpp/61.08.0012 19703352

[B3] Ashour M. L. El-Readi M. Z. Hamoud R. Eid S. Y. El A. S. Nibret E.. (2014). Anti-infective and cytotoxic properties of *Bupleurum marginatum*. Chin. Med. 9, 4–13. 10.1186/1749-8546-9-4 24438177 PMC3901767

[B4] Ashour M. L. El-Readi M. Z. Tahrani A. Eid S. Y. Wink M. (2012). A novel cytotoxic aryltetraline lactone from *Bupleurum marginatum* (*Apiaceae*). Phytochem. Lett. 5, 387–392. 10.1016/j.phytol.2012.03.009

[B5] Banerji J. Das B. Chatterjee A. Shoolery J. N. (1984). Gadain, a lignan from Jatropha-Gossypifolia. Phytochemistry 23, 2323–2327. 10.1016/S0031-9422(00)80544-0

[B6] Budzianowski J. Skrzypczak L. Walkowiak D. (1985). Flavonoids of *Parietaria-Officinalis*. J. Nat. Prod. 48, 336–337. 10.1021/np50038a033

[B7] Chen Q. L. Wang L. Feng F. (2013). Chemical constituents from the aerial part of *Echinacea purpurea*. Zhong Yao Cai 36, 739–743. 10.13863/j.issn1001-4454.2013.05.017 24218964

[B8] Chen Q. Wu F. Wang M. Dong S. Liu Y. Lu Y.. (2016). Transcriptional profiling and miRNA-target network analysis identify potential biomarkers for efficacy evaluation of Fuzheng-Huayu formula-treated hepatitis B caused liver cirrhosis. Int. J. Mol. Sci. 17, 1–14. 10.3390/ijms17060883 27271613 PMC4926417

[B9] Chen S. Li X. Yong T. Wang Z. Su J. Jiao C.. (2017). Cytotoxic lanostane-type triterpenoids from the fruiting bodies of *Ganoderma lucidum* and their structure-activity relationships. Oncotarget 8, 10071–10084. 10.18632/oncotarget.14336 28052025 PMC5354642

[B10] Cho N. Yang H. Kim J. W. Kim Y. C. Sung S. H. (2014). Chemical constituents isolated from *Disporum viridescens* leaves and their inhibitory effect on nitric oxide production in BV2 microglial cells. Bioorg. Med. Chem. Lett. 24, 5675–5678. 10.1016/j.bmcl.2014.10.068 25467159

[B11] Clichici S. Olteanu D. Filip A. Nagy A. L. Oros A. Mircea P. A. (2016). Beneficial effects of Silymarin after the discontinuation of CCl4-induced liver fibrosis. J. Med. Food 19, 789–797. 10.1089/jmf.2015.0104 27441792

[B12] Costa J. M. Telehin D. Munteanu M. Kobryn T. Ngo Y. Thibault V.. (2011). HCV-GenoFibrotest: a combination of viral, liver and genomic (IL28b, ITPA, UGT1A1) biomarkers for predicting treatment response in patients with chronic hepatitis C. Clin. Res. Hepatol. Gastroenterol. 35, 204–213. 10.1016/j.clinre.2011.01.005 21354889

[B13] Dercksen M. Duran M. IJlst L. Kulik W. Ruiter J. P. van Cruchten A.. (2016). A novel UPLC-MS/MS based method to determine the activity of N-acetylglutamate synthase in liver tissue. Mol. Genet. Metab. 119, 307–310. 10.1016/j.ymgme.2016.10.004 27771289

[B14] Di L. Yan G. Q. Wang L. Y. Ma W. Wang K. J. Li N. (2013). Two new neolignans from *Patrinia scabra* with potent cytotoxic activity against HeLa and MNK-45 cells. Arch. Pharm. Res. 36, 1198–1203. 10.1007/s12272-013-0101-y 23737105

[B15] Ding J. K. Fujino H. Kasai R. Fujimoto N. Tanaka O. Zhou J. (1986). Chemical evaluation of bupleurum species collected in Yunnan, China. Chem. Pharm. Bull. 34, 1158–1167. 10.1248/cpb.34.1158

[B16] Dong Q. Qiu L. L. Zhang C. E. Chen L. H. Feng W. W. Ma L. N.. (2016). Identification of compounds in an anti-fibrosis Chinese medicine (Fufang Biejia Ruangan Pill) and its absorbed components in rat biofluids and liver by UPLC-MS. J. Chromatogr. B Analyt. Technol. Biomed. Life Sci. 1026, 145–151. 10.1016/j.jchromb.2015.12.024 26724854

[B17] Du B. You S. (2001). Present situation in preventing and treating liver fibrosis with TCM drugs. J. Trad. Chin. Med. 21, 147–152. 11498907

[B18] Fan B. Yu Y. Zhang Y. (2015). PI3K-Akt1 expression and its significance in liver tissues with chronic fluorosis. Int. J. Clin. Exp. Pathol. 8, 1226–1236. 25973007 PMC4396260

[B19] Fan J. Li X. Li P. Li N. Wang T. Shen H.. (2007). Saikosaponin-d attenuates the development of liver fibrosis by preventing hepatocyte injury. Biochem. Cell Biol. 85, 189–195. 10.1139/O07-010 17534399

[B20] Guo Q. Zheng K. Fan D. Zhao Y. Li L. Bian Y.. (2017). Wu-Tou decoction in rheumatoid arthritis: integrating network pharmacology and *in vivo* pharmacological evaluation. Front. Pharmacol. 8:230. 10.3389/fphar.2017.00230 28515692 PMC5414545

[B21] Heinhuis B. Koenders M. I. van den Berg W. B. Netea M. G. Dinarello C. A. Joosten L. A. (2012). Interleukin 32 (IL-32) contains a typical alpha-helix bundle structure that resembles focal adhesion targeting region of focal adhesion kinase-1. J. Biol. Chem. 287, 5733–5743. 10.1074/jbc.M111.288290 22203669 PMC3285345

[B22] Hou F. Liu R. Liu X. Cui L. Wen Y. Yan S.. (2016). Attenuation of liver fibrosis by herbal compound 861 via upregulation of BMP-7/Smad signaling in the bile duct ligation model rat. Mol. Med. Rep. 13, 4335–4342. 10.3892/mmr.2016.5071 27035233

[B23] Hu Q. N. Deng Z. Tu W. Yang X. Meng Z. B. Deng Z. X.. (2014). VNP: interactive visual network pharmacology of diseases, targets, and drugs. CPT Pharmacometr. Syst. Pharmacol. 3:e105. 10.1038/psp.2014.1 24622768 PMC4039393

[B24] Iredale J. P. Pellicoro A. Fallowfield J. A. (2017). Liver Fibrosis: understanding the dynamics of bidirectional wound repair to inform the design of markers and therapies. Dig. Dis. 35, 310–313. 10.1159/000456581 28467988

[B25] Ishii H. Nakamura M. Seo S. Tori K. Tozyo T. Yoshimura Y. (1980). Isolation, characterization, and nuclear magnetic-resonance spectra of new saponins from the roots of *Bupleurum-falcatum* L. Chem. Pharm. Bull. 28, 2367–2373. 10.1248/cpb.28.2367

[B26] Kim S. Y. Yun-Choi H. S. (2007). Platelet anti-aggregating activities of bupleurumin from the aerial parts of *Bupleurum falcatum*. Arch. Pharm. Res. 30, 561–564. 10.1007/BF02977649 17615674

[B27] Kumar P. Smith T. Rahman K. Mells J. E. Thorn N. E. Saxena N. K.. (2014). Adiponectin modulates focal adhesion disassembly in activated hepatic stellate cells: implication for reversing hepatic fibrosis. FASEB J. 28, 5172–5183. 10.1096/fj.14-253229 25154876 PMC6190967

[B28] Li N. Wu J. Hasegawa T. Sakai J. Wang L. Kakuta S.. (2006). Bioactive dibenzylbutyrolactone and dibenzylbutanediol lignans from Peperomiaduclouxii. J. Nat. Prod. 69, 234–239. 10.1021/np050417o 16499322

[B29] Liang Z. T. Qin M. J. Wang Z. T. (2003). Study on the constituents of the roots of *Bupleurum marginatum*. J. China Pharm. Univ. 34, 305–308.

[B30] Liu X. Shi Y. Deng Y. Dai R. (2017a). Using molecular docking analysis to discovery dregea sinensis hemsl. potential mechanism of anticancer, antidepression, and immunoregulation. Pharmacog. Mag. 13, 358–362. 10.4103/pm.pm_384_16 28839357 PMC5551350

[B31] Liu X. Su J. Shi Y. Guo Y. Suheryani I. Zhao S.. (2017b). Herbal Formula, Baogan Yihao (BGYH), prevented Dimethylnitrosamine(DMN)-induced liver injury in rats. Drug Dev. Res. 78, 155–163. 10.1002/ddr.21388 28524372

[B32] Liu Y. Zhang T. Zhon J. Wang Q. (2008). Three new arylnaphthalide lignans from the aerial parts of *Bupleurum marginatum* WALL. ex DC. Helv. Chim. Acta 91, 2316–2320. 10.1002/hlca.200890252

[B33] Lopez H. Valera A. Trujillo J. (1996). Lignans from *Bupleurum handiense*. J. Nat. Prod. 59, 493–494. 10.1021/np960133r

[B34] Lu W. Yang G. Y. Du S. M. Wang Y. H. (2016). Advances in studies on chemical constituents and their pharmacological effect in *Bupleurum marginatum* Wall.ex DC. Herald Med. 35, 164–168.

[B35] Luetkemeyer A. F. Wyles D. L. (2017). CROI 2017: Highlights of advances in viral hepatitis and liver fibrosis. Top. Antivir. Med. 25, 84–92. 28598793 PMC5677046

[B36] Luo S. Q. Lin L. Z. Cordell G. A. (1993). Lignan glucosides from *Bupleurum-wenchuanense*. Phytochemistry 33, 193-196. 10.1016/0031-9422(93)85421-M 7764030

[B37] Mikaya G. A. Turabelidze D. G. Kemertelidze E. P. Wulfson N. S. (1981). Kaerophyllin, a new lignan from *Chaerophyllum maculatum*. Planta Med. 43, 378–380. 10.1055/s-2007-971527 17402063

[B38] Mori F. Miyase T. Ueno A. (1994). Oleanane-triterpene saponins from Clinopodium chinense var. parviflorum. Phytochemistry 36, 1485–1488. 10.1016/S0031-9422(00)89747-2 7765434

[B39] Mullen G. E. Yet L. (2015). Progress in the development of fatty acid synthase inhibitors as anticancer targets. Bioorg. Med. Chem. Lett. 25, 4363–4369. 10.1016/j.bmcl.2015.08.087 26364942

[B40] Nugraha A. S. Hilou A. Vandegraaff N. Rhodes D. I. Haritakun R. Keller P. A. (2015). Bioactive glycosides from the African medicinal plant *Boerhavia erecta* L. Nat. Prod. Res. 29, 1954–1958. 10.1080/14786419.2015.1013470 25699473

[B41] Oró D. Yudina T. Fernandez-Varo G. Casals E. Reichenbach V. Casals G.. (2016). Cerium oxide nanoparticles reduce steatosis, portal hypertension and display anti-inflammatory properties in rats with liver fibrosis. J. Hepatol. 64, 691–698. 10.1016/j.jhep.2015.10.020 26519601

[B42] Peng B. Huang X. Nakayasu E. S. Petersen J. R. Qiu S. Almeida I. C.. (2013). Using immunoproteomics to identify alpha-enolase as an autoantigen in liver fibrosis. J. Proteome Res. 12, 1789–1796. 10.1021/pr3011342 23458688 PMC3743961

[B43] Prestigiacomo V. Weston A. Messner S. Lampart F. Suter-Dick L. (2017). Pro-fibrotic compounds induce stellate cell activation, ECM-remodelling and Nrf2 activation in a human 3D-multicellular model of liver fibrosis. PLoS ONE 12:e179995. 10.1371/journal.pone.0179995 28665955 PMC5493342

[B44] Raffauf R. F. Kelley C. J. Ahmad Y. Le Quesne P. W. (1987). Alpha–and beta-peltatin from *Eriope macrostachya*. J. Nat. Prod. 50, 772–773. 10.1021/np50052a043 3430175

[B45] Sakamura S. Terayama Y. Kawakatsu S. Ichihara A. Saito H. (1980). Conjugated serotonins and phenolic constituents in safflower seed (*Carthamus-tinctorius* L). Agric. Biol. Chem. 44, 2951–2954. 10.1271/bbb1961.44.2951

[B46] Sanfeliciano A. Delcorral J. Gordaliza M. Castro A. (1991). Acidic and phenolic lignans from *Juniperus-sabina*. Phytochemistry 30, 3483–3485. 10.1016/0031-9422(91)83240-L

[B47] Sasaki T. Li W. Zaike S. Asada Y. Li Q. Ma F.. (2013). Antioxidant lignoids from leaves of *Ribes nigrum*. Phytochemistry 95, 333–340. 10.1016/j.phytochem.2013.07.022 23958345

[B48] Shimizu K. Amagaya S. Ogihara Y. (1985). New derivatives of saikosaponins. Chem. Pharm. Bull. 33, 3349–3355. 10.1248/cpb.33.3349

[B49] Sumida Y. Nakashima T. Yoh T. Nakajima Y. Ishikawa H. Mitsuyoshi H.. (2000). Serum thioredoxin levels as an indicator of oxidative stress in patients with hepatitis C virus infection. J. Hepatol. 33, 616–622. 10.1016/S0168-8278(00)80013-6 11059866

[B50] Tabet E. Genet V. Tiaho F. Lucas-Clerc C. Gelu-Simeon M. Piquet-Pellorce C.. (2016). Chlordecone potentiates hepatic fibrosis in chronic liver injury induced by carbon tetrachloride in mice. Toxicol. Lett. 255, 1–10. 10.1016/j.toxlet.2016.02.005 26853152

[B51] Tan L. Zhang Q. Y. Li J. S. Wang B. Tu G. Z. Zhao Y. Y. (2005). Studies on lignan glycosides from the roots of *Bupleurum scorzonerifolium*. Yao Xue Xue Bao 40, 428–431. 16220786

[B52] Tanemura K. Ohtaki T. Kuwahara Y. Tsumagari S. (2017). Association between liver failure and hepatic UDP-glucuronosyltransferase activity in dairy cows with follicular cysts. J. Veterin. Med. Sci. 79, 86–91. 10.1292/jvms.15-0674 27666462 PMC5289243

[B53] Trautwein C. Friedman S. L. Schuppan D. Pinzani M. (2015). Hepatic fibrosis: concept to treatment. J. Hepatol. 62, S15–S24. 10.1016/j.jhep.2015.02.039 25920084

[B54] Umezawa T. Davin L. B. Lewis N. G. (1991). Formation of lignans (-)-secoisolariciresinol and (-)-matairesinol with Forsythia intermedia cell-free extracts. J. Biol. Chem. 266, 10210–10217. 2037574

[B55] Vilarinho S. Sari S. Mazzacuva F. Bilguvar K. Esendagli-Yilmaz G. Jain D.. (2016). ACOX2 deficiency: a disorder of bile acid synthesis with transaminase elevation, liver fibrosis, ataxia, and cognitive impairment. Proc. Natl. Acad. Sci. U.S.A. 113, 11289–11293. 10.1073/pnas.1613228113 27647924 PMC5056113

[B56] Wang Q. Xu Z. L. Wang N. H. Shi Y. Y. (2007). Chemical constituents of aerial part of *Bupleurum marginatum*. J. Plant Resour. Env. 16, 71–73.

[B57] Wu X. M. Wu C. F. (2015). Network pharmacology: a new approach to unveiling traditional chinese medicine. Chin. J. Nat. Med. 13, 1–2. 10.1016/S1875-5364(15)60001-2 25660283

[B58] Yang H. Chen Y. Xu R. Shen W. Chen G. (2000). Clinical observation on the long-term therapeutic effects of traditional Chinese medicine for treatment of liver fibrosis. J. Trad. Chin. Med. 20, 247–250. 11263273

[B59] Yang Y. N. Huang X. Y. Feng Z. M. Jiang J. S. Zhang P. C. (2015). New butyrolactone type lignans from arctii fructus and their anti-inflammatory activities. J. Agric. Food Chem. 63, 7958–7966. 10.1021/acs.jafc.5b02838 26312555

[B60] Yu W. Li Z. Long F. Chen W. Geng Y. Xie Z.. (2017). A systems pharmacology approach to determine active compounds and action mechanisms of xipayi kuijie'an enema for treatment of ulcerative colitis. Sci. Rep. 7, 1–16. 10.1038/s41598-017-01335-w 28446747 PMC5430631

[B61] Zhang W. Luo J. Kong L. (2013). Chemical constituents from *Boschniakia himalaica*. Biochem. Syst. Ecol. 49, 47–50. 10.1016/j.bse.2013.03.002

[B62] Zhao X. K. Yu L. Cheng M. L. Che P. Lu Y. Y. Zhang Q.. (2017). Focal adhesion kinase regulates hepatic stellate cell activation and liver fibrosis. Sci. Rep. 7:4032. 10.1038/s41598-017-04317-0 28642549 PMC5481439

[B63] Zhou Y. N. Sun M. Y. Mu Y. P. Yang T. Ning B. B. Ren S. (2014). Xuefuzhuyu decoction inhibition of angiogenesis attenuates liver fibrosis induced by CCl(4) in mice. J. Ethnopharmacol. 153, 659–666. 10.1016/j.jep.2014.03.019 24637190

[B64] Zoubek M. E. Trautwein C. Strnad P. (2017). Reversal of liver fibrosis: from fiction to reality. Best Pract. Res. Clin. Gastroenterol. 31, 129–141. 10.1016/j.bpg.2017.04.005 28624101

