# Supplementary figures and images for: Bupleurum marginatum Wall.ex DC in Liver Fibrosis: Pharmacological Evaluation, Differential Proteomics, and Network Pharmacology
Source: Front Pharmacol. 2018 May 17;9:524. doi: 10.3389/fphar.2018.00524 (PMC5968385; doi:10.3389/fphar.2018.00524)

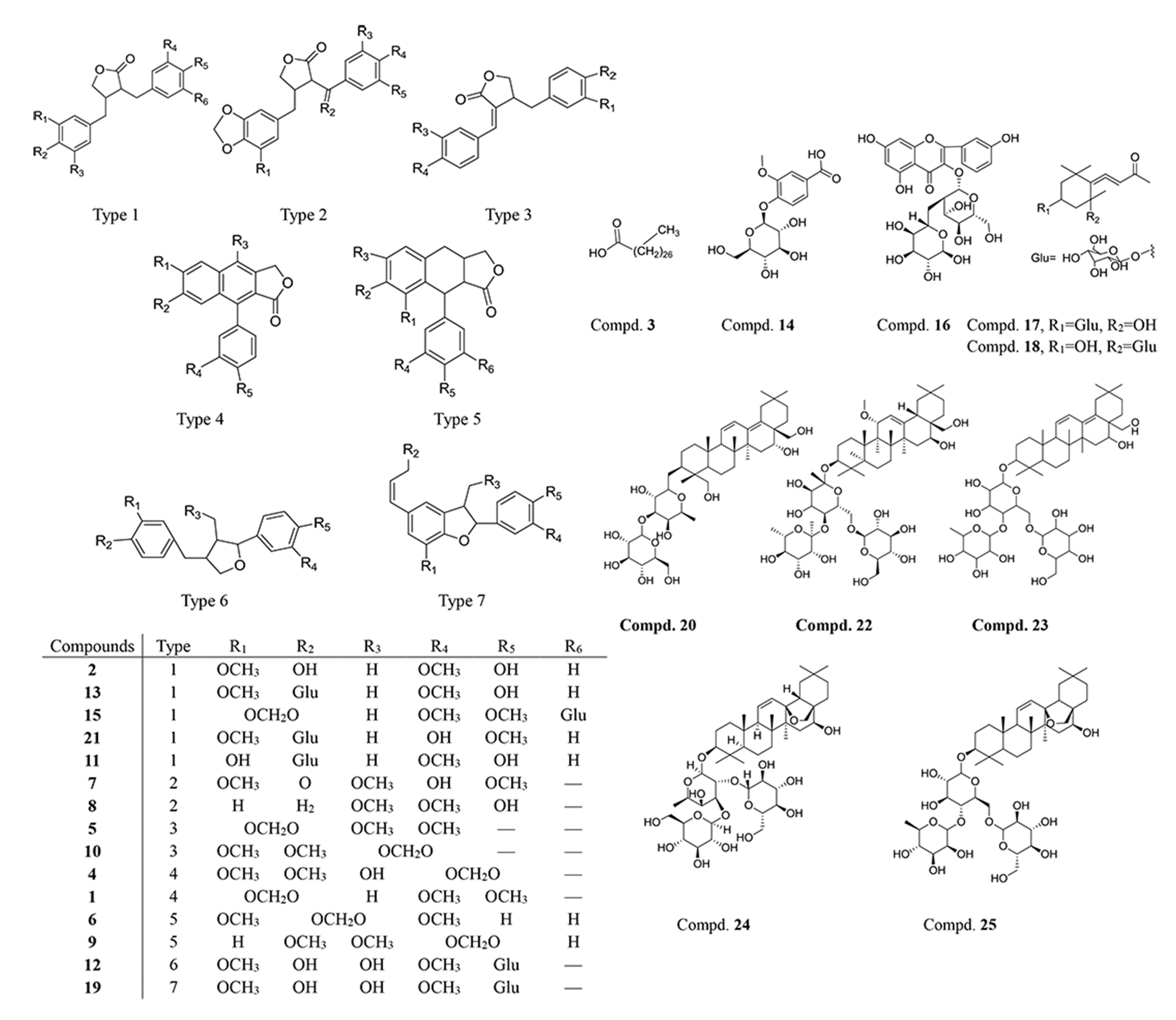

Supplement: Supplementary Figure 1 — Structures of compounds 1-25 from ZYCH. [file Image_1.JPEG]

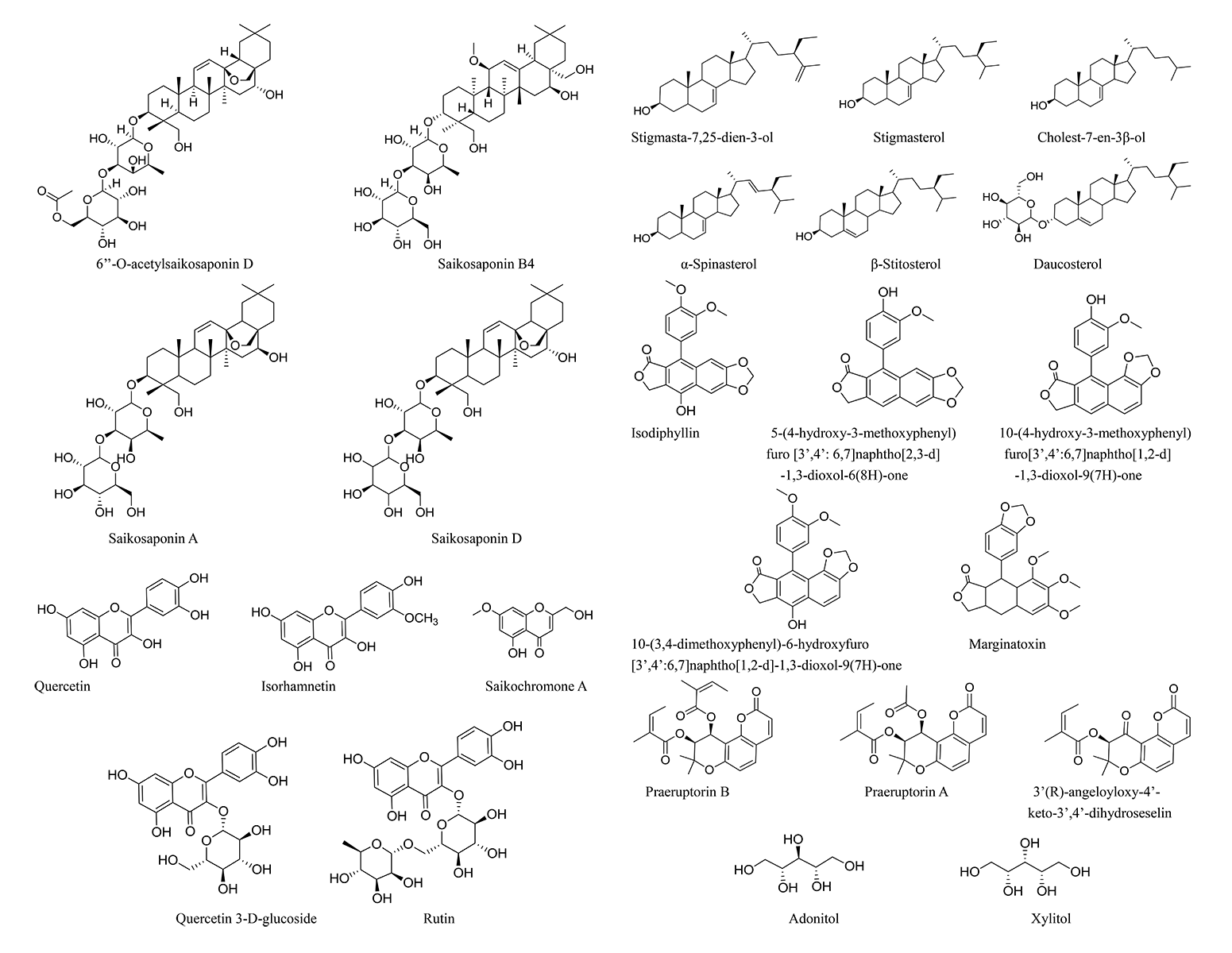

Supplement: Supplementary Figure 2 — Structures of the reported compounds from ZYCH in literatures. [file Image_2.TIF]

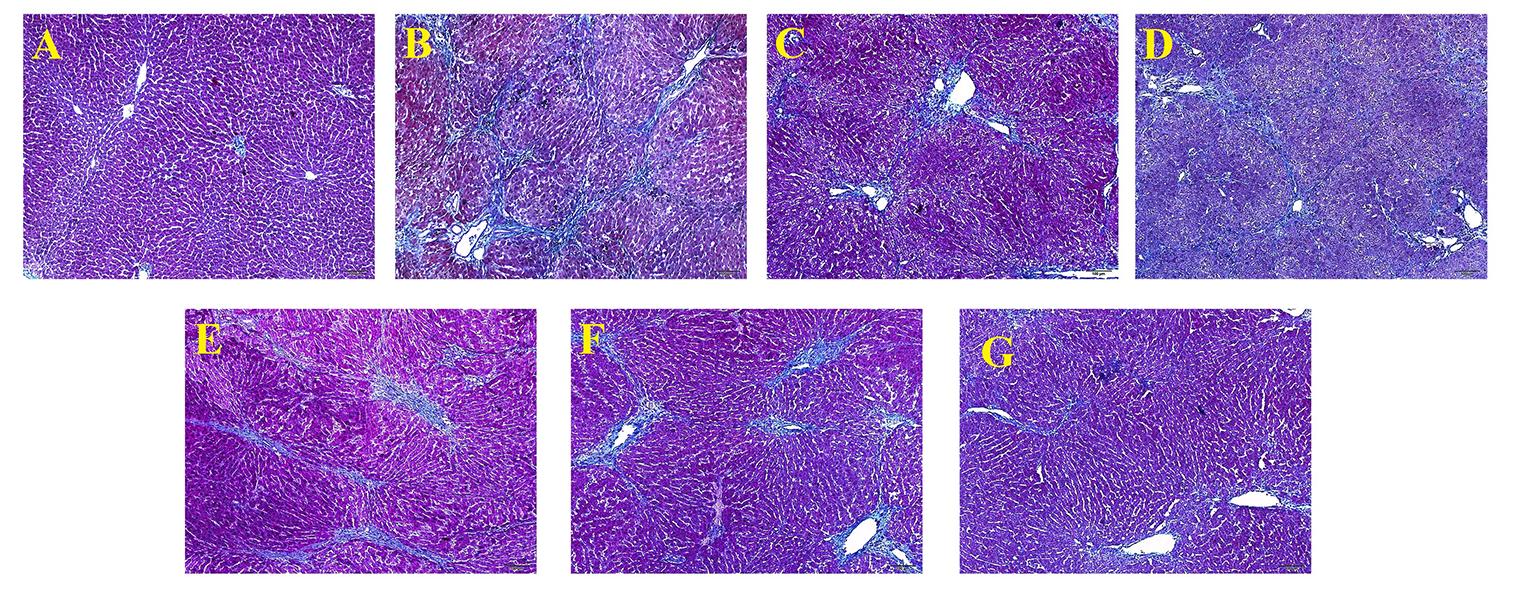

Supplement: Supplementary Figure 3 — Effects of ZYCH on Masson's trichrome staining changes of DMN-induced liver injury rats. Original magnification: 100×. (A) Control group, (B) model group, (C) SC group, (D) L-ZYCH group, (E) M-ZYCH group, (F) H-ZYCH group, and (G) HR-ZYCH group. [file Image_3.TIF]

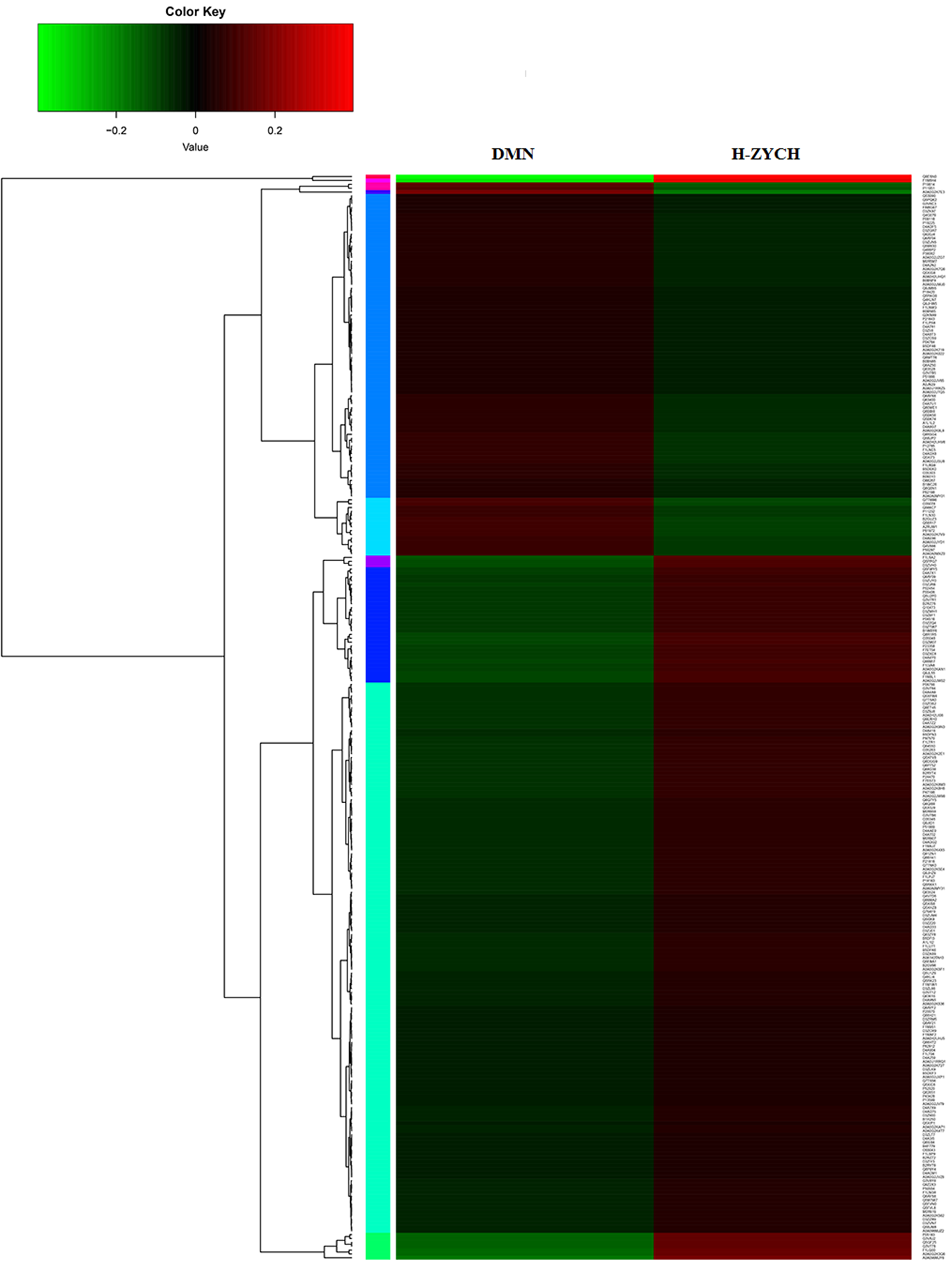

Supplement: Supplementary Figure 4 — Hierarchical cluster analysis of differentially expressed proteins between H-ZYCH and DMN-model groups. The rows represent each protein and the column means different groups. The color in the graph indicates the relative expression of the protein in the sample, red represents the higher expression level and the green represents the lower expression. The left independent column represents the classification of the protein (10 classes). [file Image_4.TIF]

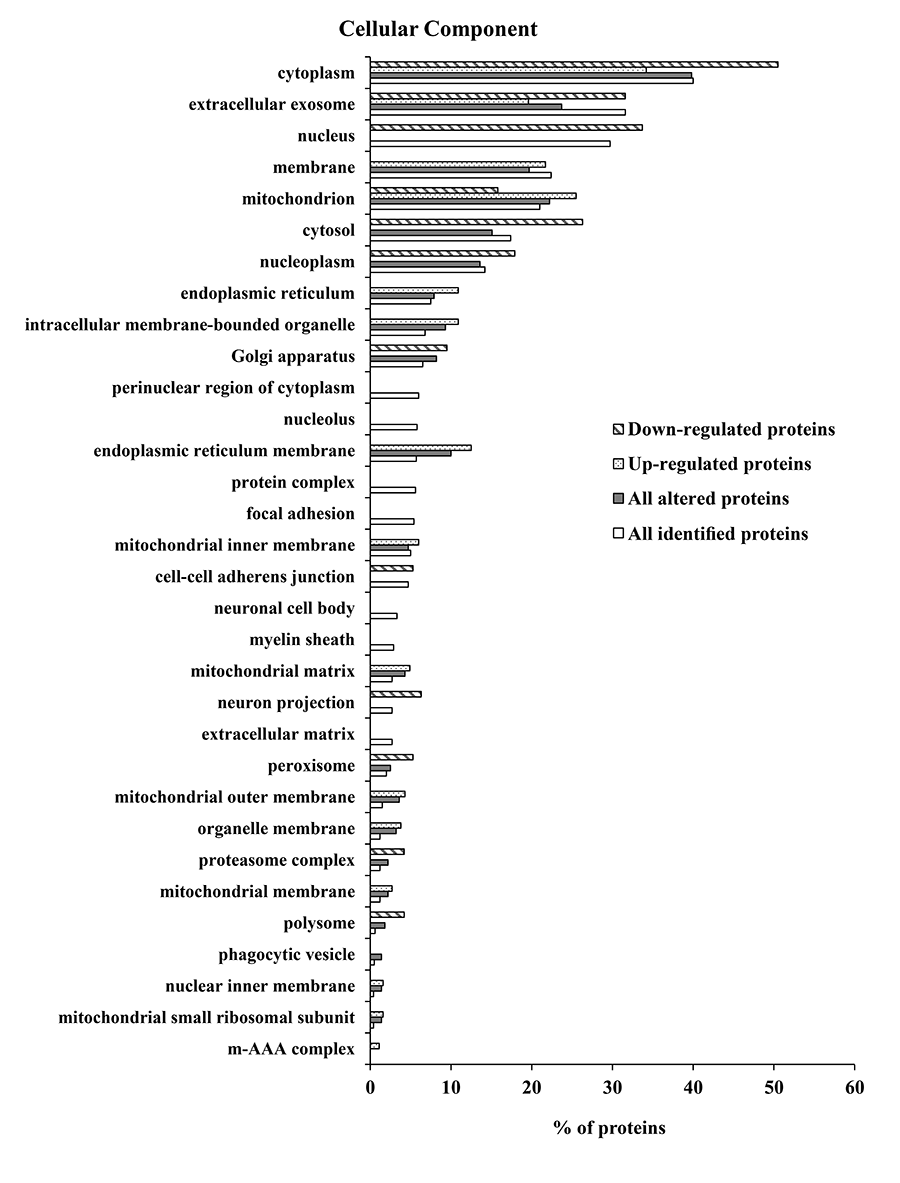

Supplement: Supplementary Figure 5 — GO classification of all identified proteins, all altered proteins, up-regulated altered proteins and down-regulated altered proteins between H-ZYCH and DMN-model groups in cellular component. Union of The first 15 subtypes of all identified proteins and all altered proteins in GO classification were analyzed. [file Image_5.TIF]

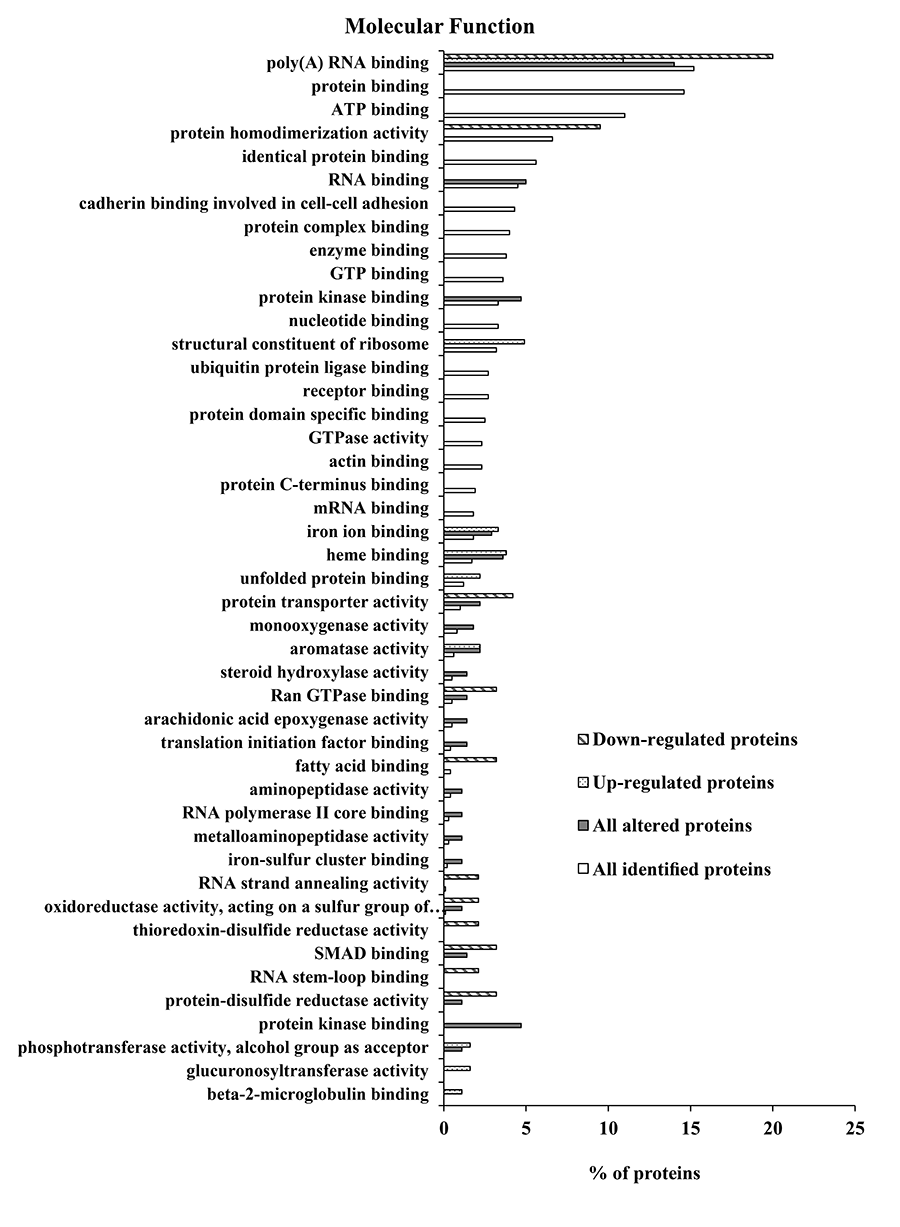

Supplement: Supplementary Figure 6 — GO classification of all identified proteins, all altered proteins, up-regulated altered proteins and down-regulated altered proteins between H-ZYCH and DMN-model groups in molecular function. Union of The first 15 subtypes of all identified proteins and all altered proteins in GO classification were analyzed. [file Image_6.TIF]

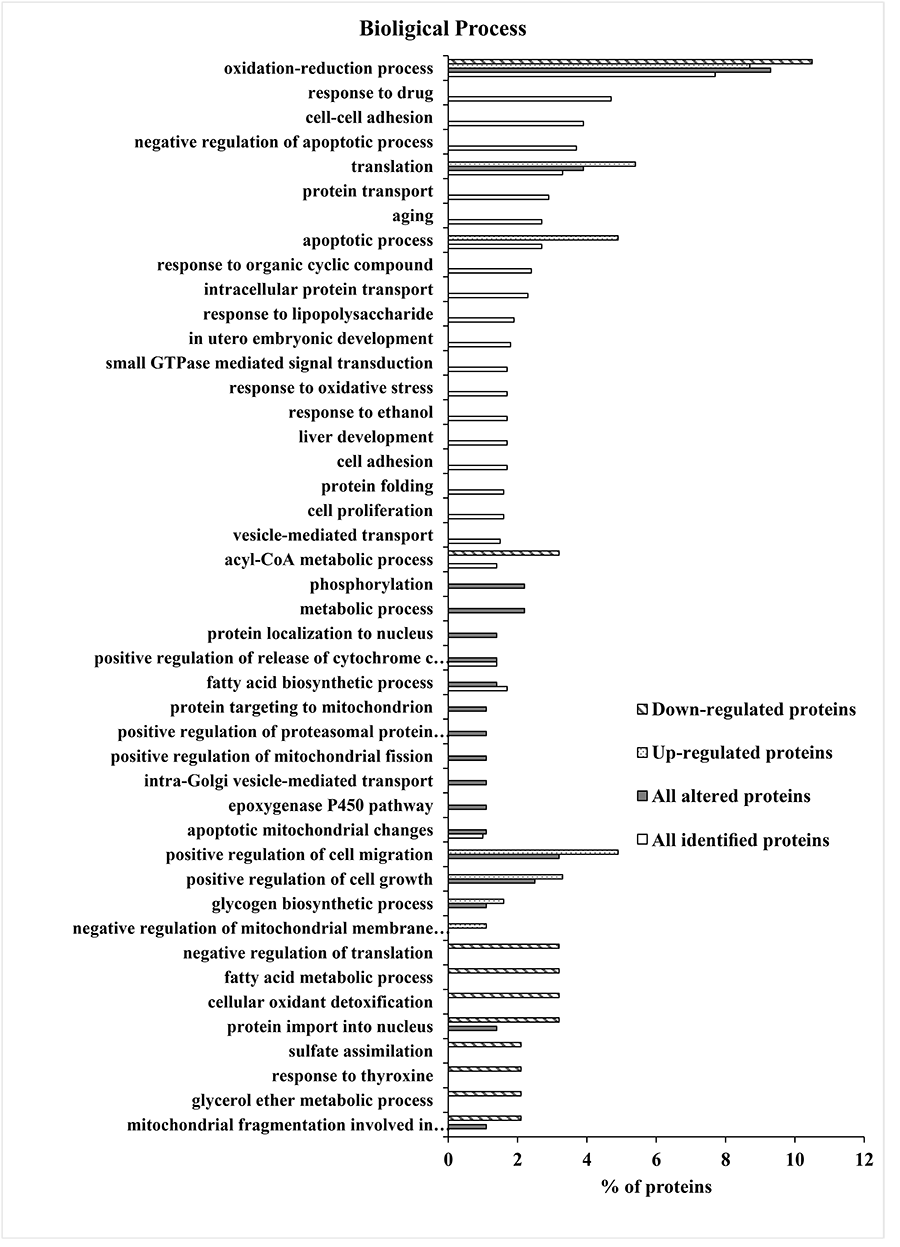

Supplement: Supplementary Figure 7 — GO classification of all identified proteins, all altered proteins, up-regulated altered p6roteins and down-regulated altered proteins between H-ZYCH and DMN-model groups in biological process. Union of The first 15 subtypes of all identified proteins and all altered proteins in GO classification were analyzed. [file Image_7.TIF]

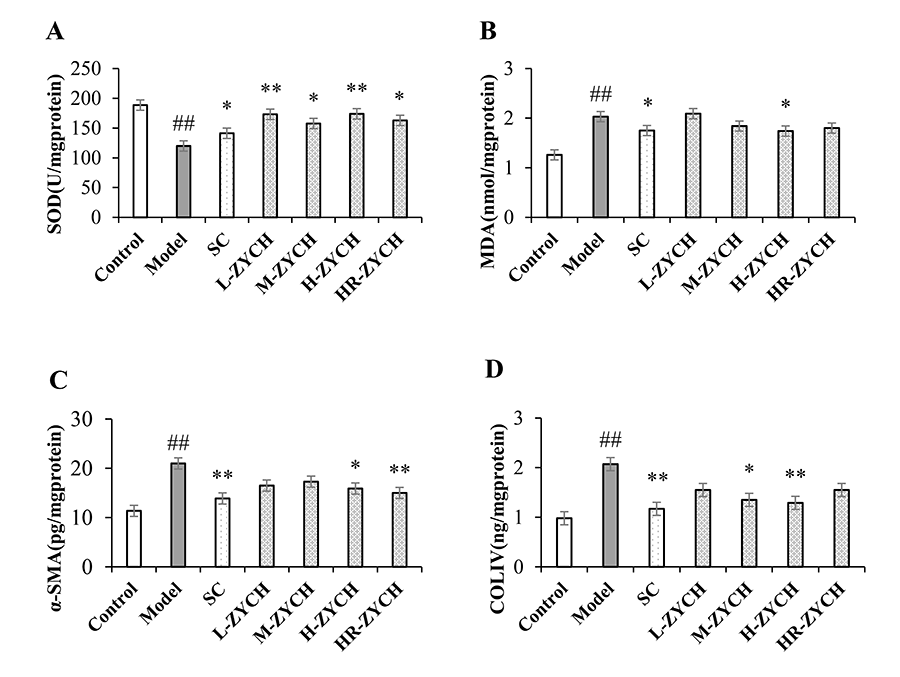

Supplement: Supplementary Figure 8 — ELISA analysis for proteomics validation of H-ZYCH and DMN-model rats in liver tissues. (A) The activity of SOD; (B) the production of MDA; (C) the production of α-SMA; (D) the production of COLIV. #p < 0.05, ##p < 0.01 vs. control group, *p < 0.05, **p < 0.01 vs. model group. [file Image_8.TIF]
