# Supplementary material for: Bupleurum marginatum Wall.ex DC in Liver Fibrosis: Pharmacological Evaluation, Differential Proteomics, and Network Pharmacology
Source: Front Pharmacol. 2018 May 17;9:524. doi: 10.3389/fphar.2018.00524 (PMC5968385; doi:10.3389/fphar.2018.00524)
Supplement: Supplementary Table 1 — The experimental schedule of ZYCH anti-liver fibrosis induced by DMN. [file Table_1.DOCX]

supplementary Table 1. The experimental schedule of ZYCH anti-liver fibrosis induced by DMN.

| group | treatment | | after-treatment |
| --- | --- | --- | --- |
|  | injection | gavage |  |
| control group  Control | intraperitoneally injected saline，0.1mL/100g，3d/week | saline, 1mL/100g/d | After  4 weeks, the rats were sacrificed under anaesthesia. |
| DMN model group  Model | intraperitoneally injected 1% DMN，0.1mL/100g，3d/week | saline, 1mL/100g/d |  |
| positive control group  SC | intraperitoneally injected 1% DMN，0.1mL/100g，3d/week | 5mg/mL silymarin capsules  1mL/100g/d（0.05g/Kg） |  |
| low-dose ZYCH group  L-ZYCH | intraperitoneally injected 1% DMN，0.1mL/100g，3d/week | 25mg/mL ZYCH  1mL/100g/d（0.25g/Kg） |  |
| middle-dose ZYCH group  M-ZYCH | intraperitoneally injected 1% DMN，0.1mL/100g，3d/week | 50 mg/mL ZYCH  1mL/100g/d（0.5g/Kg） |  |
| high-dose ZYCH group  H-ZYCH | intraperitoneally injected 1% DMN，0.1mL/100g，3d/week | 100mg/mL ZYCH  1mL/100g/d（1.0g/Kg） |  |
| higher-dose ZYCH group  HR-ZYCH | intraperitoneally injected 1% DMN，0.1mL/100g，3d/week | 200mg/mL ZYCH  1mL/100g/d（2.0g/Kg） |  |
