# Supplementary material for: Bupleurum marginatum Wall.ex DC in Liver Fibrosis: Pharmacological Evaluation, Differential Proteomics, and Network Pharmacology
Source: Front Pharmacol. 2018 May 17;9:524. doi: 10.3389/fphar.2018.00524 (PMC5968385; doi:10.3389/fphar.2018.00524)
Supplement: Supplementary Table 2 — The molecular docking results of ZYCH anti-liver fibrosis protein targets. [file Table_2.DOCX]

Supplementary table 2. The molecular docking results of ZYCH anti-liver fibrosis protein targets

| Protein IDs | Protein names | gene | PDB IDs | regulation | Target compounds |
| --- | --- | --- | --- | --- | --- |
| GABT_RAT | 4-aminobutyrate aminotransferase, mitochondrial | Abat | 4Y0I | up | LG-indigoticalignanoside A(12) |
| ACOT1_RAT | Acyl-coenzyme A thioesterase 1 | Acot1 | 5SZV | down | S-Saikosaponin C(25)  S-Salikosaponin A  S-clinoposaponin XI(24)  LG-dehydrodiconiferyl alcohol 4-O-β-D-glucopyranoside(19)  LG-styraxlignolide C(21)  FA-Octacosanoic acid(3)  L-butyrolactone(7)  LG-(-)-matairesinol 4-O-glucoside(13) |
| F8WG67_RAT | Acyl-CoA thioesterase 7, isoform CRA_a | Acot7 | 2V1O | down | LG-indigoticalignanoside A(12)  S-Saikosaponin C(25)  S-Salikosaponin A  STG-Daucosterin  FA-Octacosanoic acid(3)  LG-matairesinol monoglucoside(11)  LG-dehydrodiconiferyl alcohol 4-O-β-D-glucopyranoside(19)  S-clinoposaponin XI(24)  LG-(-)-matairesinol 4-O-glucoside(13) |
| F1LNW3_RAT | Acyl-coenzyme A oxidase | Acox2 | 1W07 | down | STG-Daucosterin  FA-Octacosanoic acid(3)  LG-styraxlignolide C(21)  LG-dehydrodiconiferyl alcohol 4-O-β-D-glucopyranoside(19)  L-(-)-matairesinol(2)  L-(-)-matairesinol 4-O-glucoside(13) |
| ACSL1_RAT | Long-chain-fatty-acid--CoA ligase 1 | Acsl1 | 3G7S | up | FA-Octacosanoic acid(3) |
| F1M1W1_RAT | Acyl-CoA synthetase medium-chain family member 1 | Acsm1 | 3DAY | up | S-Saikosaponin C(25)  S-Salikosaponin A  LG-dehydrodiconiferyl alcohol 4-O-β-D-glucopyranoside(19)  S-clinoposaponin XI(24)  LG-(-)-matairesinol 4-O-glucoside(13)  LG-indigoticalignanoside A(12)  LG-matairesinol monoglucoside(11)  S-Salikosaponin H(23)  FA-Octacosanoic acid(3) |
| Q7TMB6_RAT | Acyl-coenzyme A synthetase ACSM5, mitochondrial | Acsm5 | 5JRH | down | S-Saikosaponin C(25)  LG-indigoticalignanoside A(12)  STG-Daucosterin  S-Salikosaponin A  S-clinoposaponin XI(24)  LG-styraxlignolide C(21)  FA-Octacosanoic acid(3)  LG-matairesinol monoglucoside(11)  LG-(-)-matairesinol 4-O-glucoside(13) |
| A0A0G2K562_RAT | Disintegrin and metalloproteinase domain-containing protein 10 | Adam10 | 5L0Q | up | FA-Octacosanoic acid(3)  LG-styraxlignolide C(21)  S-Salikosaponin A  S-clinoposaponin XI(24) |
| A0A0G2JW98_RAT | Alcohol dehydrogenase 1 | Adh1 | 5ENV | up | FA-Octacosanoic acid(3)  L-(-)-matairesinol(2)  L-(-)-matairesinol 4-O-glucoside(13) |
| G3V784_RAT | ADP-dependent glucokinase | Adpgk | 4B8R | up | S-Saikosaponin C(25)  LG-styraxlignolide C(21)  FA-Octacosanoic acid(3)  S-clinoposaponin XI(24)  STG-Daucosterin  LG-indigoticalignanoside A(12)  LG-dehydrodiconiferyl alcohol 4-O-β-D-glucopyranoside(19)  L-butyrolactone(7)  LG-matairesinol monoglucoside(11) |
| AIP_RAT | AH receptor-interacting protein | Aip | 4APO | up | S-Saikosaponin C(25)  FA-Octacosanoic acid(3)  L-butyrolactone(7)  LG-(-)-matairesinol 4-O-glucoside(13)  S-clinoposaponin XI(24)  FG-Quercetin-3-O-β-D-glucopyranoside  STG-Daucosterin  S-Salikosaponin A  LG-matairesinol monoglucoside(11) |
| A0A0G2K7Q6_RAT | Adenylate kinase isoenzyme 1 | Ak1 | 1Z83 | down | S-Saikosaponin B2(20)  S-Salikosaponin A  LG-styraxlignolide C(21)  S-clinoposaponin XI(24)  S-Saikosaponin C(25)  LG-matairesinol monoglucoside(11)  STG-Daucosterin  LG-dehydrodiconiferyl alcohol 4-O-β-D-glucopyranoside(19)  S-Salikosaponin H(23) |
| AKT1_RAT | RAC-alpha serine/threonine-protein kinase | Akt1 | 5KCV | up | LG-dehydrodiconiferyl alcohol 4-O-β-D-glucopyranoside(19)  FA-Octacosanoic acid(3)  LG-(-)-matairesinol 4-O-glucoside(13)  LG-indigoticalignanoside A(12)  L-butyrolactone(7)  L-α-peltatin(6) |
| A0A0G2K3Q6_RAT | Fructose-bisphosphate aldolase | Aldoc | 4TR9 | up | LG-indigoticalignanoside A(12)  LG-matairesinol monoglucoside(11)  LG-(-)-matairesinol 4-O-glucoside(13) |
| GDIR1_RAT | Rho GDP-dissociation inhibitor 1 | Arhgdia | 1KMT | down | — |
| ARHG7_RAT | Rho guanine nucleotide exchange factor 7 | Arhgef7 | 5SXP | up | FA-Octacosanoic acid(3)  S-Saikosaponin C(25)  S-clinoposaponin XI(24)  LG-indigoticalignanoside A(12)  L-butyrolactone(7)  LG-styraxlignolide C(21)  LG-dehydrodiconiferyl alcohol 4-O-β-D-glucopyranoside(19)  S-Salikosaponin H(23) |
| ATG3_RAT | Ubiquitin-like-conjugating enzyme ATG3 | Atg3 | 2DYT | down | — |
| F1LU71_RAT | AU RNA binding protein/enoyl-coenzyme A hydratase | Auh | 4OMR | up | FA-Octacosanoic acid(3)  LT-(+)-3'-Angeloyloxy-4'-keto-3',4'-dihydroseselin  L-bupleurumin(9)  LG-matairesinol monoglucoside(11)  LG-indigoticalignanoside A(12) |
| CYBP_RAT | Calcyclin-binding protein | Cacybp | 1X5M | down | — |
| CBR4_RAT | Carbonyl reductase family member 4 | Cbr4 | 4CQM | up | S-clinoposaponin XI(24)  FA-Octacosanoic acid(3)  LG-indigoticalignanoside A(12)  LG-dehydrodiconiferyl alcohol 4-O-β-D-glucopyranoside(19)  LG-matairesinol monoglucoside(11)  LG-(-)-matairesinol 4-O-glucoside(13) |
| CDO1_RAT | Cysteine dioxygenase type 1 | Cdo1 | 5I0U | up | FA-Octacosanoic acid(3)  LG-styraxlignolide C(21)  LG-indigoticalignanoside A(12)  LG-dehydrodiconiferyl alcohol 4-O-β-D-glucopyranoside(19)  LG-matairesinol monoglucoside(11)  STG-Daucosterin |
| D4AAE9_RAT | CDGSH iron sulfur domain 2 | Cisd2 | 4OOA | up | FA-Octacosanoic acid(3)  LG-dehydrodiconiferyl alcohol 4-O-β-D-glucopyranoside(19)  LG-indigoticalignanoside A(12)  L-(-)-matairesinol(2)  L-(-)-matairesinol 4-O-glucoside(13)  LG-styraxlignolide C(21) |
| D4A8T3_RAT | Coatomer protein complex, subunit zeta 1 | Copz1 | 5MC7 | down | — |
| COX2_RAT | Cytochrome c oxidase subunit 2 | COX2 | 2YEV | up | FA-Octacosanoic acid(3)  LG-dehydrodiconiferyl alcohol 4-O-β-D-glucopyranoside(19)  L-demethylyatein(8)  LG-indigoticalignanoside A(12)  L-(-)-matairesinol(2)  L-(-)-matairesinol 4-O-glucoside(13) |
| NB5R3_RAT | NADH-cytochrome b5 reductase 3 | Cyb5r3 | 5GV7 | up | FA-Octacosanoic acid(3)  S-Saikosaponin C(25)  LG-styraxlignolide C(21)  S-Salikosaponin A  S-Salikosaponin H(23)  LG-(-)-matairesinol 4-O-glucoside(13)  LG-matairesinol monoglucoside(11)  LG-indigoticalignanoside A(12)  LG-dehydrodiconiferyl alcohol 4-O-β-D-glucopyranoside(19) |
| OXDA_RAT | D-amino-acid oxidase | Dao | 4YJF | down | LG-indigoticalignanoside A(12)  LG-matairesinol monoglucoside(11)  FA-Octacosanoic acid(3)  LG-styraxlignolide C(21)  L-butyrolactone(7)  FG-Quercetin-3-O-β-D-glucopyranoside  LG-(-)-matairesinol 4-O-glucoside(13) |
| A0A0G2K719_RAT | DEAD-box helicase 3, X-linked | Ddx3x | 5E7I | down | FA-Octacosanoic acid(3)  ST-Cholest-7-en-3β-ol |
| DNM1L_RAT | Dynamin-1-like protein | Dnm1l | 3W6O | down | L-butyrolactone(7)  LT-(+)-3'-Angeloyloxy-4'-keto-3',4'-dihydroseselin |
| TOR1A_RAT | Torsin-1A | Dyt1 | 5J1S | up | S-clinoposaponin XI(24)  LG-indigoticalignanoside A(12)  LG-styraxlignolide C(21)  S-Salikosaponin H(23)  S-Salikosaponin A  LG-matairesinol monoglucoside(11)  FA-Octacosanoic acid(3)  L-butyrolactone(7)  S-Saikosaponin C(25) |
| ECH1_RAT | Delta(3,5)-Delta(2,4)-dienoyl-CoA isomerase, mitochondrial | Ech1 | 1DCI | up | FA-Octacosanoic acid(3) |
| Q5RKG9_RAT | Eukaryotic translation initiation factor 4B | Eif4b | 1WI8 | down | — |
| ENOA_RAT | Alpha-enolase | Eno1 | 2PSN | down | — |
| A0A0A0MY01_RAT | Fatty acid-binding protein, intestinal | Fabp2 | 3AKN | down | LG-matairesinol monoglucoside(11)  LG-styraxlignolide C(21)  FA-Octacosanoic acid(3)  LG-dehydrodiconiferyl alcohol 4-O-β-D-glucopyranoside(19)  LG-(-)-matairesinol 4-O-glucoside(13)  STG-Daucosterin  LG-indigoticalignanoside A(12)  LT-Praeruptorin A |
| FAS_RAT | Fatty acid synthasesynthase | Fasn | 5C37 | down | FA-Octacosanoic acid(3)  L-α-peltatin(6)  LG-dehydrodiconiferyl alcohol 4-O-β-D-glucopyranoside(19)  LG-indigoticalignanoside A(12)  ST-Cholest-7-en-3β-ol |
| FCGRN_RAT | IgG receptor FcRn large subunit p51 | Fcgrt | 4K71 | up | FA-Octacosanoic acid(3)  LG-dehydrodiconiferyl alcohol 4-O-β-D-glucopyranoside(19) |
| D3ZZR9_RAT | Peptidylprolyl isomerase | Fkbp2 | 4J4O | up | S-Saikosaponin C(25)  S-clinoposaponin XI(24)  FA-Octacosanoic acid(3)  LG-indigoticalignanoside A(12)  S-Salikosaponin A  LG-styraxlignolide C(21)  L-butyrolactone(7) |
| A0A0G2JTQ5_RAT | N-acetylgalactosamine kinase | Galk2 | 2A2C | down | L-butyrolactone(7)  SBG-asicariside b1(17)  L-bupleurumin(9)  L-demethylyatein(8) |
| GALT1_RAT | Polypeptide N-acetylgalactosaminyltransferase 1 | Galnt1 | 1XHB | up | FA-Octacosanoic acid(3)  LG-styraxlignolide C(21)  S-Salikosaponin A  LG-matairesinol monoglucoside(11)  S-clinoposaponin XI(24)  S-Saikosaponin C(25) |
| A0A096MJY6_RAT | 1,4-alpha-glucan-branching enzyme 1 | Gbe1 | 5E6Z | up | FA-Octacosanoic acid(3)  ST-Stigmasta-7,25-dien-3β-ol |
| GUAD_RAT | Guanine deaminase | Gda | 4AQL | down | F-saikochromone A |
| GDE1_RAT | Glycerophosphodiester phosphodiesterase 1 | Gde1 | 3CH0 | up | S-Saikosaponin C(25)  LG-matairesinol monoglucoside(11)  FA-Octacosanoic acid(3)  S-Salikosaponin A  LG-indigoticalignanoside A(12) |
| D3ZUM4_RAT | Beta-galactosidase | Glb1 | 5MUJ | up | LG-indigoticalignanoside A(12) |
| Q5BK56_RAT | Glutathione S-transferase | Gstm4 | 5J41 | down | S-clinoposaponin XI(24)  S-Saikosaponin C(25)  LG-dehydrodiconiferyl alcohol 4-O-β-D-glucopyranoside(19)  L-butyrolactone(7)  LG-indigoticalignanoside A(12)  LG-(-)-matairesinol 4-O-glucoside(13)  FA-Octacosanoic acid(3) |
| A0A0G2JXP1_RAT | Glycogenin-1 | Gyg1 | 3U2U | up | S-clinoposaponin XI(24)  S-Saikosaponin B2(20)  STG-Daucosterin  S-Saikosaponin C(25)  LG-indigoticalignanoside A(12)  S-Salikosaponin A  FA-Octacosanoic acid(3)  LG-matairesinol monoglucoside(11)  LG-styraxlignolide C(21) |
| B0BNF9_RAT | Hydroxyacid oxidase 1 | Hao1 | 2RDU | down | L-(-)-matairesinol(2)  L-(-)-matairesinol 4-O-glucoside(13) |
| DHB11_RAT | Estradiol 17-beta-dehydrogenase 11 | Hsd17b11 | 1YB1 | up | FA-Octacosanoic acid(3)  LG-matairesinol monoglucoside(11)  LG-dehydrodiconiferyl alcohol 4-O-β-D-glucopyranoside(19)  L-bupleurumin(9) |
| D4A2N2_RAT | Inositol polyphosphate-5-phosphatase B | Inpp5b | 2XSW | down | FG-Quercetin-3-O-β-D-glucopyranoside  LG-matairesinol monoglucoside(11)  FA-Octacosanoic acid(3)  LG-styraxlignolide C(21)  LG-indigoticalignanoside A(12)  LG-dehydrodiconiferyl alcohol 4-O-β-D-glucopyranoside(19)  S-clinoposaponin XI(24) |
| D4A781_RAT | Importin 5 | Ipo5 | 5WUN | down | FA-Octacosanoic acid(3)  S-clinoposaponin XI(24)  S-Saikosaponin C(25)  S-Salikosaponin A  LG-indigoticalignanoside A(12)  LG-styraxlignolide C(21)  LG-matairesinol monoglucoside(11)  STG-Daucosterin  LG-(-)-matairesinol 4-O-glucoside(13) |
| A0A0A0MXZ0_RAT | Iron-sulfur cluster assembly 1 homolog, mitochondrial | Isca1 | 1X0G | down | F-saikochromone A |
| A0A0G2JVZ6_RAT | Integrin subunit alpha V | Itgav | 4WK0 | up | L-α-peltatin(6) |
| A0A0A0MY31_RAT | Inositol 1,4,5-trisphosphate receptor type 1 | Itpr1 | 1XZZ | up | S-Saikosaponin C(25)  FA-Octacosanoic acid(3)  LG-styraxlignolide C(21)  LG-dehydrodiconiferyl alcohol 4-O-β-D-glucopyranoside(19)  S-Salikosaponin A  LG-(-)-matairesinol 4-O-glucoside(13)  S-clinoposaponin XI(24)  LG-indigoticalignanoside A(12) |
| Q56R17_RAT | Importin subunit alpha | Kpna4 | 4RXH | down | — |
| F1M8J7_RAT | Homeobox protein cut-like 1 | LOC681658 | 1X2L | up | — |
| Q499P2_RAT | Leukotriene A(4) hydrolase | Lta4h | 5BPP | down |  |
| A0A0G2K5F1_RAT | LRP16 protein | Macrod1 | 2X47 | up | FA-Octacosanoic acid(3)  S-Salikosaponin A  LG-indigoticalignanoside A(12)  L-α-peltatin(6) |
| MANEA_RAT | Glycoprotein endo-alpha-1,2-mannosidase | Manea | 4ACY | up | FA-Octacosanoic acid(3)  S-Saikosaponin C(25)  LG-matairesinol monoglucoside(11)  LG-dehydrodiconiferyl alcohol 4-O-β-D-glucopyranoside(19)  ST-7-Sitoster-3β-ol  LG-indigoticalignanoside A(12)  ST-Cholest-7-en-3β-ol  LG-(-)-matairesinol 4-O-glucoside(13)  ST-Stigmasta-7,25-dien-3β-ol |
| F1LRB8_RAT | S-adenosylmethionine synthase | Mat2a | 5T8S | down | — |
| MAP2_RAT | Methionine aminopeptidase 2 | Metap2 | 1B6A | down | FA-Octacosanoic acid(3)  LG-styraxlignolide C(21)  LG-matairesinol monoglucoside(11)  L-butyrolactone(7)  LG-indigoticalignanoside A(12)  L-demethylyatein(8)  PA-vanillic acid 4-β-d-glucoside(14)  LG-dehydrodiconiferyl alcohol 4-O-β-D-glucopyranoside(19) |
| B2GUZ3_RAT | Methylenetetrahydrofolate dehydrogenase | Mthfd1l | 1DIA | down | FA-Octacosanoic acid(3)  LG-indigoticalignanoside A(12)  LG-styraxlignolide C(21)  S-Saikosaponin C(25)  S-clinoposaponin XI(24)  LG-dehydrodiconiferyl alcohol 4-O-β-D-glucopyranoside(19)  L-butyrolactone(7) |
| D4A904_RAT | N-acetylglutamate synthase | Nags | 4K30 | up | FA-Octacosanoic acid(3)  L-butyrolactone(7)  FG-Quercetin-3-O-β-D-glucopyranoside  L-(-)-matairesinol(2)  L-(-)-matairesinol 4-O-glucoside(13) |
| D3ZLK9_RAT | ATP-dependent (S)-NAD(P)H-hydrate dehydratase | Naxd | 3RPZ | up | LG-styraxlignolide C(21)  LG-matairesinol monoglucoside(11)  STG-Daucosterin  LG-(-)-matairesinol 4-O-glucoside(13)  S-Saikosaponin C(25)  FA-Octacosanoic acid(3)  LG-indigoticalignanoside A(12) |
| A0A0U1RRQ1_RAT | NADH:ubiquinone oxidoreductase subunit A1 | Ndufa1 | 4G74 | up | LG-dehydrodiconiferyl alcohol 4-O-β-D-glucopyranoside(19)  LG-(-)-matairesinol 4-O-glucoside(13)  FA-Octacosanoic acid(3)  STG-Daucosterin  LG-styraxlignolide C(21)  S-Salikosaponin A  L-butyrolactone(7)  LG-indigoticalignanoside A(12) |
| A0A0H2UI06_RAT | NADH dehydrogenase (ubiquinone) complex I, assembly factor 6 | Ndufaf6 | 5JWC | up | S-Saikosaponin C(25)  FA-Octacosanoic acid(3)  L-butyrolactone(7)  LG-indigoticalignanoside A(12)  LG-matairesinol monoglucoside(11)  S-Salikosaponin A |
| NUDT3_RAT | Diphosphoinositol polyphosphate phosphohydrolase 1 | Nudt3 | 2Q9P | down | S-Saikosaponin C(25)  S-clinoposaponin XI(24)  STG-Daucosterin  LG-indigoticalignanoside A(12)  S-Salikosaponin A  S-Saikosaponin B2(20)  LG-matairesinol monoglucoside(11)  LG-styraxlignolide C(21)  LG-(-)-matairesinol 4-O-glucoside(13) |
| NTF2_RAT | Nuclear transport factor 2 | Nutf2 | 5BXQ | down | S-Saikosaponin C(25)  LG-(-)-matairesinol 4-O-glucoside(13)  LG-styraxlignolide C(21)  FA-Octacosanoic acid(3)  LG-matairesinol monoglucoside(11)  LG-indigoticalignanoside A(12)  STG-Daucosterin  S-Salikosaponin A  S-clinoposaponin XI(24) |
| PA1B3_RAT | Platelet-activating factor acetylhydrolase IB subunit gamma | Pafah1b3 | 3DT8 | up | S-Salikosaponin H(23)  FA-Octacosanoic acid(3)  LG-matairesinol monoglucoside(11)  LG-indigoticalignanoside A(12)  S-clinoposaponin XI(24)  LG-styraxlignolide C(21)  STG-Daucosterin  LG-dehydrodiconiferyl alcohol 4-O-β-D-glucopyranoside(19) |
| SYPM_RAT | Probable proline--tRNA ligase, mitochondrial | Pars2 | 5IFU | up | FA-Octacosanoic acid(3)  S-Saikosaponin C(25)  LG-styraxlignolide C(21)  LG-matairesinol monoglucoside(11)  L-butyrolactone(7) |
| B5DF46_RAT | Phosphomannomutase | Pmm2 | 2I55 | down | LG-indigoticalignanoside A(12)  FA-Octacosanoic acid(3)  L-α-peltatin(6)  L-butyrolactone(7)  S-Saikosaponin C(25)  LG-dehydrodiconiferyl alcohol 4-O-β-D-glucopyranoside(19)  L-(-)-matairesinol(2)  LG-matairesinol monoglucoside(11) |
| D3ZUN5_RAT | Protein O-fucosyltransferase 2 | Pofut2 | 5FOE | down | FA-Octacosanoic acid(3)  LG-indigoticalignanoside A(12)  LG-styraxlignolide C(21) |
| DPOLB_RAT | DNA polymerase beta | Polb | 5V1N | up | S-Saikosaponin C(25)  S-Salikosaponin A  FA-Octacosanoic acid(3)  LG-dehydrodiconiferyl alcohol 4-O-β-D-glucopyranoside(19)  LG-styraxlignolide C(21)  LG-matairesinol monoglucoside(11) |
| Q4KLI4_RAT | Peptidyl-prolyl cis-trans isomerase | Ppil1 | 5LUD | up | S-clinoposaponin XI(24)  S-Saikosaponin C(25)  S-Salikosaponin H(23)  LG-(-)-matairesinol 4-O-glucoside(13)  LG-styraxlignolide C(21)  FA-Octacosanoic acid(3)  S-Salikosaponin A  LG-matairesinol monoglucoside(11)  LG-dehydrodiconiferyl alcohol 4-O-β-D-glucopyranoside(19) |
| D3ZVN7_RAT | Protoporphyrinogen oxidase | Ppox | 3NKS | up | FA-Octacosanoic acid(3)  LG-matairesinol monoglucoside(11)  S-Salikosaponin A  LG-indigoticalignanoside A(12)  LG-styraxlignolide C(21)  LG-dehydrodiconiferyl alcohol 4-O-β-D-glucopyranoside(19)  LG-(-)-matairesinol 4-O-glucoside(13) |
| A0A0H2UHV6_RAT | Calcineurin subunit B type 1 | Ppp3r1 | 4OR9 | down | FA-Octacosanoic acid(3)  LG-dehydrodiconiferyl alcohol 4-O-β-D-glucopyranoside(19)  L-butyrolactone(7)  LG-indigoticalignanoside A(12)  LG-matairesinol monoglucoside(11)  LG-(-)-matairesinol 4-O-glucoside(13)  L-(-)-matairesinol(2)  L-(-)-matairesinol 4-O-glucoside(13) |
| G3V7B5_RAT | Phosphoribosyl pyrophosphate synthase-associated protein 1 | Prpsap1 | 2C4K | down | LG-(-)-matairesinol 4-O-glucoside(13)  LG-matairesinol monoglucoside(11)  LG-dehydrodiconiferyl alcohol 4-O-β-D-glucopyranoside(19)  LG-indigoticalignanoside A(12)  LG-styraxlignolide C(21)  FA-Octacosanoic acid(3)  L-butyrolactone(7)  L-α-peltatin(6) |
| PRS8_RAT | 26S protease regulatory subunit 8 | Psmc5 | 5L4G | down | — |
| FAK1_RAT | Focal adhesion kinase 1 | Ptk2 | 4D5H | up | FA-Octacosanoic acid(3)  LG-matairesinol monoglucoside(11)  LG-(-)-matairesinol 4-O-glucoside(13)  LG-dehydrodiconiferyl alcohol 4-O-β-D-glucopyranoside(19)  S-Saikosaponin C(25) |
| G3V7B6_RAT | Phosphatidylglycerophosphatase and protein-tyrosine phosphatase 1 | Ptpmt1 | 3RGQ | up | S-clinoposaponin XI(24)  LG-matairesinol monoglucoside(11)  S-Saikosaponin C(25)  FA-Octacosanoic acid(3)  S-Salikosaponin H(23)  LG-dehydrodiconiferyl alcohol 4-O-β-D-glucopyranoside(19)  PA-vanillic acid 4-β-d-glucoside(14)  S-11 (α) -methoxy saikosaponin F(22)  LG-styraxlignolide C(21) |
| F1M951_RAT | Tyrosine-protein phosphatase non-receptor type 23 | Ptpn23 | 5LM1 | up | FA-Octacosanoic acid(3)  LG-styraxlignolide C(21)  LG-(-)-matairesinol 4-O-glucoside(13)  S-Salikosaponin H(23)  LG-indigoticalignanoside A(12) |
| RAB43_RAT | Ras-related protein Rab-43 | Rab43 | 2HUP | down | LG-styraxlignolide C(21)  LG-matairesinol monoglucoside(11)  FA-Octacosanoic acid(3)  LG-(-)-matairesinol 4-O-glucoside(13)  STG-Daucosterin  LG-indigoticalignanoside A(12)  L-demethylyatein(8) |
| D3ZCS9_RAT | 5-hydroxyisourate hydrolase | RGD1309350 | 3Q1E | down |  |
| MIRO2_RAT | Mitochondrial Rho GTPase 2 | Rhot2 | 5KUT | up | S-clinoposaponin XI(24)  S-Saikosaponin C(25)  FA-Octacosanoic acid(3)  LG-matairesinol monoglucoside(11)  LG-(-)-matairesinol 4-O-glucoside(13) |
| RFA2_RAT | Replication protein A 32 kDa subunit | Rpa2 | 4MQV | down |  |
| B5DEK0_RAT | Regulation of nuclear pre-mRNA domain containing 1B | Rprd1b | 4HFG | down | LT-(+)-3'-Angeloyloxy-4'-keto-3',4'-dihydroseselin |
| A0A0G2JZG7_RAT | Serine--tRNA ligase, cytoplasmic | Sars | 1WLE | down | LG-matairesinol monoglucoside(11)  FA-Octacosanoic acid(3)  LG-(-)-matairesinol 4-O-glucoside(13)  LG-styraxlignolide C(21)  L-(-)-matairesinol(2)  L-(-)-matairesinol 4-O-glucoside(13)  LG-dehydrodiconiferyl alcohol 4-O-β-D-glucopyranoside(19)  LG-indigoticalignanoside A(12) |
| D3Z9J8_RAT | Spermidine/spermine N1-acetyl transferase 2 | Sat2 | 5CNP | up | S-Saikosaponin C(25)  LG-styraxlignolide C(21)  LG-matairesinol monoglucoside(11)  FA-Octacosanoic acid(3)  L-butyrolactone(7)  LG-indigoticalignanoside A(12)  S-clinoposaponin XI(24) |
| Q6P9Y4_RAT | ADP/ATP translocase 1 | Slc25a4 | 2C3E | up | LG-indigoticalignanoside A(12)  S-Salikosaponin A  LG-(-)-matairesinol 4-O-glucoside(13)  LG-styraxlignolide C(21)  LG-matairesinol monoglucoside(11)  LG-dehydrodiconiferyl alcohol 4-O-β-D-glucopyranoside(19)  FA-Octacosanoic acid(3) |
| 4F2_RAT | 4F2 cell-surface antigen heavy chain | Slc3a2 | 2DH2 | up | FA-Octacosanoic acid(3)  LG-styraxlignolide C(21)  LG-(-)-matairesinol 4-O-glucoside(13)  L-butyrolactone(7)  S-Salikosaponin A  LG-matairesinol monoglucoside(11)  LG-dehydrodiconiferyl alcohol 4-O-β-D-glucopyranoside(19) |
| MID51_RAT | Mitochondrial dynamics protein MID51 | Smcr7l | 4OAG | up | S-clinoposaponin XI(24)  L-butyrolactone(7)  FA-Octacosanoic acid(3)  LG-dehydrodiconiferyl alcohol 4-O-β-D-glucopyranoside(19)  LG-matairesinol monoglucoside(11) |
| A0A0G2K536_RAT | Paraplegin | Spg7 | 2QZ4 | up | S-Saikosaponin C(25)  LG-(-)-matairesinol 4-O-glucoside(13)  FA-Octacosanoic acid(3)  S-clinoposaponin XI(24)  LG-matairesinol monoglucoside(11)  LG-indigoticalignanoside A(12)  LG-dehydrodiconiferyl alcohol 4-O-β-D-glucopyranoside(19)  LG-styraxlignolide C(21)  S-Salikosaponin A |
| SUV3_RAT | ATP-dependent RNA helicase SUPV3L1, mitochondrial | Supv3l1 | 5SUP | up | L-chinensin(1)  L-α-peltatin(6) |
| TACO1_RAT | Translational activator of cytochrome c oxidase 1 | Taco1 | 5EKZ | up | S-Saikosaponin C(25)  S-Salikosaponin A  S-11 (α) -methoxy saikosaponin F(22)  S-Salikosaponin H(23)  LG-matairesinol monoglucoside(11)  LG-dehydrodiconiferyl alcohol 4-O-β-D-glucopyranoside(19)  FA-Octacosanoic acid(3)  LG-indigoticalignanoside A(12)  S-clinoposaponin XI(24) |
| TMED2_RAT | Transmembrane emp24 domain-containing protein 2 | Tmed2 | 5AZW | up | FA-Octacosanoic acid(3)  S-Saikosaponin C(25)  LG-styraxlignolide C(21)  S-Salikosaponin H(23)  S-Salikosaponin A  LG-indigoticalignanoside A(12)  LG-matairesinol monoglucoside(11)  STG-Daucosterin  S-clinoposaponin XI(24) |
| TOIP2_RAT | Torsin-1A-interacting protein 2 | Tor1aip2 | 5J1T | up | S-Salikosaponin H(23)  S-clinoposaponin XI(24)  S-Saikosaponin C(25)  S-Salikosaponin A  LG-indigoticalignanoside A(12)  FA-Octacosanoic acid(3)  LG-styraxlignolide C(21)  LG-matairesinol monoglucoside(11)  LG-dehydrodiconiferyl alcohol 4-O-β-D-glucopyranoside(19) |
| THIO_RAT | Thioredoxin | Txn1 | 5HR0 | down | FA-Octacosanoic acid(3)  S-Saikosaponin C(25)  L-butyrolactone(7)  S-clinoposaponin XI(24)  L-(-)-matairesinol(2)  L-(-)-matairesinol 4-O-glucoside(13)  LG-indigoticalignanoside A(12)  LG-styraxlignolide C(21)  LG-dehydrodiconiferyl alcohol 4-O-β-D-glucopyranoside(19) |
| TXNL1_RAT | Thioredoxin-like protein 1 | Txnl1 | 4YOD | down | — |
| UBA3_RAT | NEDD8-activating enzyme E1 catalytic subunit | Uba3 | 3GZN | up | LG-(-)-matairesinol 4-O-glucoside(13)  S-Saikosaponin C(25) |
| UFC1_RAT | Ubiquitin-fold modifier-conjugating enzyme 1 | Ufc1 | 3EVX | down | S-clinoposaponin XI(24)  FA-Octacosanoic acid(3)  S-Saikosaponin C(25)  LG-styraxlignolide C(21)  LG-matairesinol monoglucoside(11)  L-(-)-matairesinol(2)  L-(-)-matairesinol 4-O-glucoside(13)  LG-indigoticalignanoside A(12) |
| UD11_RAT | UDP-glucuronosyltransferase 1A1 | Ugt1a1 | 1FGG | up | S-Saikosaponin C(25)  LG-indigoticalignanoside A(12)  S-Salikosaponin A  LG-styraxlignolide C(21)  FA-Octacosanoic acid(3)  LG-dehydrodiconiferyl alcohol 4-O-β-D-glucopyranoside(19) |
| A0A0G2JW02_RAT | UDP-glucuronosyltransferase | Ugt2a3 | 2O6L | up | — |
| A0A0G2K727_RAT | UDP-glucuronosyltransferase | Ugt2b35 | 2O6L | up | FA-Octacosanoic acid(3)  LG-styraxlignolide C(21)  STG-Daucosterin  LG-dehydrodiconiferyl alcohol 4-O-β-D-glucopyranoside(19)  LG-matairesinol monoglucoside(11)  LG-(-)-matairesinol 4-O-glucoside(13)  LG-indigoticalignanoside A(12)  L-butyrolactone(7) |
| URIC_RAT | Uricase | Uox | 5FRC | down | PA-vanillic acid 4-β-d-glucoside(14)  S-Salikosaponin H(23)  FA-Octacosanoic acid(3)  LG-dehydrodiconiferyl alcohol 4-O-β-D-glucopyranoside(19)  LG-indigoticalignanoside A(12) |
| F1LPJ7_RAT | Ubiquitinyl hydrolase 1 | Usp33 | 5GG4 | up | FA-Octacosanoic acid(3)  ST-7-Sitoster-3β-ol |
| VAMP7_RAT | Vesicle-associated membrane protein 7 | Vamp7 | 4B93 | down | FA-Octacosanoic acid(3)  L-butyrolactone(7)  LG-indigoticalignanoside A(12) |
| WDR7_RAT | WD repeat-containing protein 7 | Wdr7 | 5IBK | up | S-Salikosaponin A  LG-indigoticalignanoside A(12)  LG-styraxlignolide C(21)  LG-(-)-matairesinol 4-O-glucoside(13)  FA-Octacosanoic acid(3)  S-Saikosaponin B4  STG-Daucosterin  LG-matairesinol monoglucoside(11)  S-Saikosaponin C(25) |
| XPP2_RAT | Xaa-Pro aminopeptidase 2 | Xpnpep2 | 3CTZ | up | LG-dehydrodiconiferyl alcohol 4-O-β-D-glucopyranoside(19)  FA-Octacosanoic acid(3) |
| A0A0G2JV65_RAT | 14-3-3 protein zeta/delta | Ywhaz | 5NAS | down | S-clinoposaponin XI(24)  FA-Octacosanoic acid(3)  S-Saikosaponin C(25)  S-Salikosaponin A  LG-matairesinol monoglucoside(11)  LG-dehydrodiconiferyl alcohol 4-O-β-D-glucopyranoside(19)  L-butyrolactone(7)  S-Salikosaponin H(23) |
